# Convergence of dominance and neglect in flying insect diversity

**DOI:** 10.1038/s41559-023-02066-0

**Published:** 2023-05-18

**Authors:** Amrita Srivathsan, Yuchen Ang, John M. Heraty, Wei Song Hwang, Wan F. A. Jusoh, Sujatha Narayanan Kutty, Jayanthi Puniamoorthy, Darren Yeo, Tomas Roslin, Rudolf Meier

**Affiliations:** 1 Center for Integrative Biodiversity Discovery, Leibniz Institute for Evolution and Biodiversity Science, Museum für Naturkunde, Berlin, Germany; 2 Lee Kong Chian Natural History Museum, National University of Singapore, Singapore, Singapore; 3 Department of Entomology, University of California, Riverside, CA USA; 4 School of Science, Monash University Malaysia, Subang Jaya, Malaysia; 5 Department of Biological Sciences, National University of Singapore, Singapore, Singapore; 6 Tropical Marine Science Institute, National University of Singapore, Singapore, Singapore; 7 Department of Ecology, Swedish University of Agricultural Sciences, Uppsala, Sweden

**Keywords:** Entomology, Biodiversity

## Abstract

Most of arthropod biodiversity is unknown to science. Consequently, it has been unclear whether insect communities around the world are dominated by the same or different taxa. This question can be answered through standardized sampling of biodiversity followed by estimation of species diversity and community composition with DNA barcodes. Here this approach is applied to flying insects sampled by 39 Malaise traps placed in five biogeographic regions, eight countries and numerous habitats (>225,000 specimens belonging to >25,000 species in 458 families). We find that 20 insect families (10 belonging to Diptera) account for >50% of local species diversity regardless of clade age, continent, climatic region and habitat type. Consistent differences in family-level dominance explain two-thirds of variation in community composition despite massive levels of species turnover, with most species (>97%) in the top 20 families encountered at a single site only. Alarmingly, the same families that dominate insect diversity are ‘dark taxa’ in that they suffer from extreme taxonomic neglect, with little signs of increasing activities in recent years. Taxonomic neglect tends to increase with diversity and decrease with body size. Identifying and tackling the diversity of ‘dark taxa’ with scalable techniques emerge as urgent priorities in biodiversity science.

## Abstract (web summary)

Using DNA barcoding to analyse flying insect diversity of >225,000 specimens from five biogeographic regions, the authors show that more than half of local species diversity is represented by only 20 insect families, most of which suffer from taxonomic neglect.

## Main

Biodiversity loss is now widely recognized as a major threat to planetary health^1–3^. Halting the loss requires that the basic building blocks of biodiversity are known, so that changes can be recorded, drivers of change can be identified and appropriate policy actions can be implemented. However, much of the terrestrial animal diversity belongs to hyperdiverse invertebrate clades that are so poorly known^4,5^ that it is difficult to obtain this critical information. For example, only 0.17 G of the 2.16 G records in the Global Biodiversity Information Facility pertain to arthropods. By comparison, 67% of Global Biodiversity Information Facility records relate to birds, although birds account for only 10,000–20,000 species (0.2%) of the estimated 8–10 million multicellular species worldwide^6,7^. These numbers alone reveal the size of the knowledge gap for many truly diverse clades that due to their current position in the information shadow have been called ‘dark taxa’^8^.

To allocate resources for discovering and conserving species, it is crucial to establish the relative contribution of different taxa to overall biodiversity. Only in this way can the most diverse and abundant taxa be given adequate attention. Identifying these taxa is furthermore important for understanding the basic structure of the living world, and for gaining insights into how community composition is shaped by evolutionary, biogeographic or ecological factors^9^. Where such analyses have been carried out—for example, for plants and snakes^10^—they have revealed that a few clades dominate communities across the world^11^. Unfortunately, corresponding information is lacking for arthropods. This is a striking shortcoming, given that arthropods are found worldwide, functionally important^12^ and currently undergoing major declines in diversity and abundance^13,14^.

In this Article, we analyse the taxonomic patterns among flying insects sampled by Malaise traps in different habitats, climates and biogeographic regions. Malaise traps are widely used in global biomonitoring programmes because they provide standardized and efficient tools for collecting diverse communities of flying insects and semi-aquatic taxa^15–17^. Similar to all other trap types, they only subsample the insect communities. For example, Malaise traps rely on the passive interception of insect flight paths, and collect those insects that climb towards the highest point of the trap (Supplementary Fig. 1). For this reason, strong and active fliers like dragonflies (which largely avoid the traps) or beetles (which tend to drop to the ground when encountering an obstacle) are under-represented. However, overall, Malaise traps are so effective at sampling flying insects that sample processing is a major challenge due to high specimen and species yields^15,18^. In addition, most specimens caught in Malaise traps cannot be identified, because many species are undescribed and relevant taxonomic expertise is either non-existent or dwindling^6^. Fortunately, recent advances in large-scale DNA barcoding with new sequencing technologies allow for processing large numbers of specimens rapidly and cost-effectively^19,20^. Using molecular species delimitation methods, these data can then be converted into estimates of species diversity without formal description of the component taxa and most species can be assigned to major insect clades for analysis of community structure.

We here determine the taxonomic composition of Malaise trap samples^21^ from five biogeographic regions, eight countries and diverse habitats. In total, our material encompasses >225,000 specimens belonging to >25,000 species living in habitats ranging from temperate meadows to tropical rainforests. We discover surprising congruence with regard to which 20 insect families are dominant components of flying arthropod communities worldwide (accounting for >50% of species and specimens in each sample). When we compare family-specific diversity with taxonomic attention, we find that most of the particularly diverse and abundant taxa are poorly known and suffer from persistent taxonomic neglect. In other words, a very large proportion of terrestrial animal biodiversity is not only unknown to science, but will also remain so for the foreseeable future unless such ‘dark taxa’ become a preferred target for biodiversity science.

## Results

**Fig. 1:**
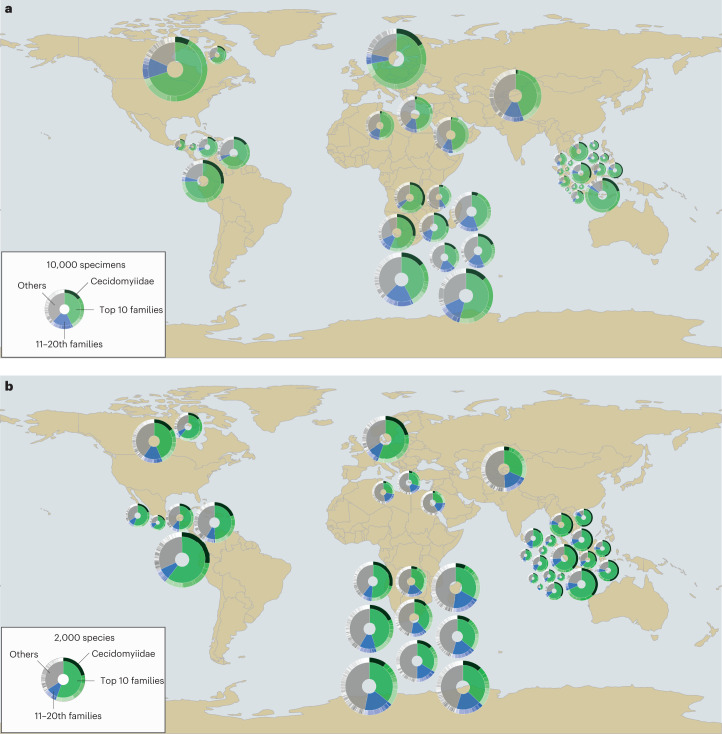
Congruence in the relative contribution of insect families to specimen abundance and species richness. **a**,**b**, Each chart shows the taxonomic composition of a sample obtained by an individual Malaise trap at a specific site. The inner circle represents the proportion of biodiversity in the top 10 (green), next 10 (blue) and remaining families (grey). The outer ring shows what proportion of biodiversity belongs to the top 10, next 10 and the remaining families. Black is used to illustrate the extraordinary diversity of Cecidomyiidae (Diptera). All charts are scaled relative to number of specimens (**a**) and species (**b**) at each site. Map made with Natural Earth. Supplementary Fig. 2 provides precise geolocations for each site.

**Fig. 2:**
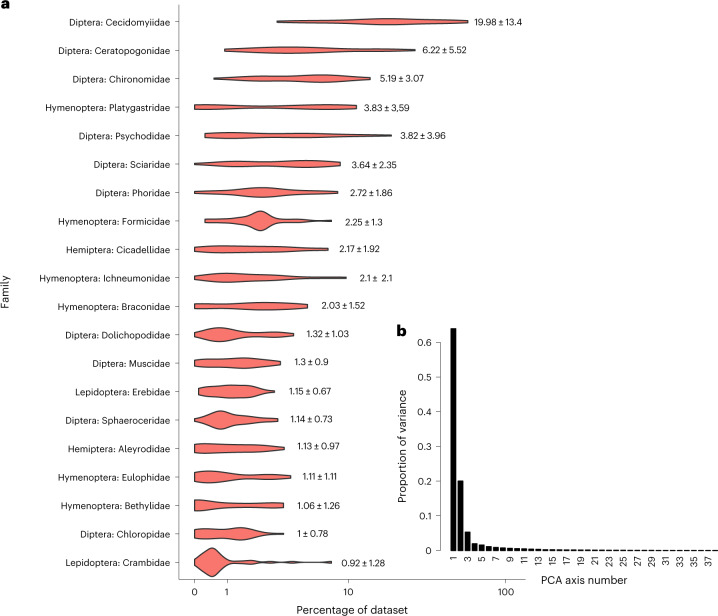
Proportional species richness of top 20 insect families in Malaise traps. **a**,**b**, Consistency in community composition of insects as shown by proportional (%) species richness of the top 20 families among sites (*x*-axis log-transformed) (**a**) and proportion of variance absorbed by the first axis in a PCA of variation in the proportion of species richness per insect family (**b**). Individual violins in the violin plot in **a** show the average proportional contribution ± standard deviation for each individual family.

Our study comprises 225,261 barcoded arthropods belonging to 458 families. They represent the insect diversity obtained from 39 traps across eight different sites and all continents excluding Australia and Antarctica. Applying a species-delimitation threshold of 3% sequence similarity^20,22^ reveals that each Malaise trap yielded anywhere from 69 to 3,426 molecular operational taxonomic units (mOTUs). When these mOTUs were assigned to higher arthropod clades, we found that, on average, 57.2% and 19.0% of the species in a trap belonged to the orders Diptera and Hymenoptera (Supplementary Fig. 3.1). When examined at the family level, 61.7% of the specimens and 51.9% of the species in each trap belong to a set of only ten insect families (henceforth referred to as the ‘top 10 families’). The next ten families added only 9.7% and 12.2% of specimens and species, respectively (Fig. 1: see the ‘top 20 families’). Nearly one-fifth of the species per site (average 20%) belonged to a single dipteran family, Cecidomyiidae (Fig. 2a).

The relative species richness of individual insect families showed remarkably similar patterns across the globe, with the top 20 families (Fig. 1b) accounting for 41.2–72.3% of the total species richness regardless of continent or climate (Canada: 63.9%, Egypt: 47.3%, Germany: 65.8%, Honduras: 65.5%, South Africa: 54.5%, Pakistan: 49.4%, Saudi Arabia: 41.2% and Singapore: 72.3%). Yet, the high species richness of these families was not correlated with clade age (*r* = 0.0039; *n* = 10,023, *P* = 0.70, Supplementary Fig. 5).

The dominance of specific insect families across sampling sites is perhaps best illustrated by the consistent differences in species richness among families. In our main dataset (39 Malaise traps), 66.4% of the variation among traps in log-transformed species richness was explained by family. These results persisted irrespective of the species delimitation method used (with an adjusted *R*^*2*^ of 66.4% when species were delimited by objective clustering^23^ at a 3% distance threshold, and an adjusted *R*^*2*^ of 64.9% when species were delimited by Assemble Species by Automatic Partitioning (ASAP)^24^). In our expanded dataset (Methods), which retained sample-level resolution for traps placed in Germany and Canada (56 datasets), the corresponding figure was 67.1% for objective clustering at 3%. The only qualitative difference in results between the main and expanded datasets was whether Mycetophilidae (Diptera) and Crambidae (Lepidoptera) were included among the top 20 families. Note that this list of top 20 families is furthermore robust to changes in family designations. This was tested by merging clades with their sister clades based on recent phylogenies to thereby account for taxonomists’ disagreements on rank (Supplementary Table 3). Nineteen of the top 20 families remain in the list, and the only change involved the replacement of one lepidopteran clade (Gelechiidae + Cosmopterigidae replaced Crambidae). Unsurprisingly, the convergence of taxonomic composition was even stronger at the order level (90.6% of log-transformed species richness based on adjusted *R*^2^ and objective clustering at 3.0% distance threshold, Supplementary Fig. 3.1).

The convergence of relative species richness was also evident from a principal component analysis (PCA), which revealed that >60% of the variance can be explained by a single principal component (Fig. 2b and Supplementary Figs. 3.2b and 3.3b). In contrast, analysis of species turnover between the major regions showed that almost all species in the top 20 families (97.6%) were found at a single site only (Supplementary Table 2). In other words, variation in community composition was largely attributable to variation in the relative contribution of distinct species from a small set of families.

**Fig. 3:**
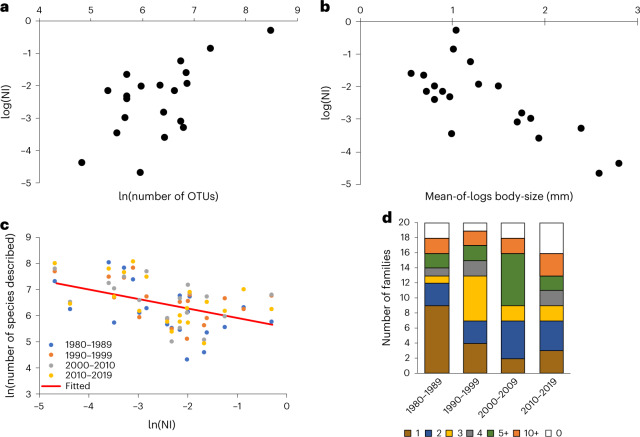
Taxonomic neglect, species diversity and taxonomic activity dedicated to the top 20 families. **a**,**b**, Taxonomic neglect (as expressed by the NI) increases with the species diversity of the target taxon (**a**), and taxonomic neglect decreases with increasing body size of the target taxon (**b**). **c**, The more neglected a taxon is, the less taxonomic attention is dedicated to it (with no sign of improvement over time). **d**, Likewise, the number of authors publishing monographic work on the top 20 families shows no increase over time. The stacked bars show the proportion of families with 0 (white), 1 (brown), 2 (blue), 3 (yellow), 4 (grey), 5–9 (green) and 10+ (orange) authors having described ≥50 species in a decade (see also Supplementary Fig. 4).

Given the disproportionate contribution of a few families to insect diversity across the world, we next examined whether the globally dominant taxa have attracted appropriate taxonomic attention. To characterize taxonomic attention or neglect, we first defined a ‘neglect index’ (NI) as the ratio between the number of mOTUs found across the Malaise traps for a given family and the total number of species described as listed in the Catalog of Life (CoL: https://www.catalogueoflife.org/). An NI value of 1 signals that we detected as many mOTUs in the current set of 39 Malaise traps as have been formally described for the entire world, whereas a low NI value reveals that we found only a tiny proportion of all species described so far. We then investigated how this index is correlated with species richness and body size. We found a positive correlation between the log NI and the log number of mOTUs detected in our samples (main dataset: *r* = 0.61, *n* = 20, *P* = 0.004; expanded dataset: *r* = 0.54, *n* = 20, *P* = 0.01) (Fig. 3 and Supplementary Fig. 4). Moreover, we saw a strong negative relationship between the NI and the number of species described per decade between 1980 and 2019 (main dataset, *r* = 0.44, *n* = 80, *P* = 0.00003; expanded dataset, *r* = 0.48, *n* = 80, *P* = 7.6 × 10^−6^). In other words, the more neglected a taxon is, the fewer new species are described per decade.

Furthermore, we see no signs of any increase in taxonomic attention paid to neglected taxa over time: the slope of the relationship between species richness and taxonomic neglect showed no detectable change over time (non-significant interaction decade × NI for the main dataset; analysis of variance (ANOVA): *F*_6,72_ = 0.86, *P* = 0.53, expanded dataset, ANOVA; *F*_6,72_ = 0.80, *P* = 0.58). In a similar vein, the number of taxonomists involved in monographic work (that is, the number of taxonomists describing ≥50 species in a decade) shows no increase over time (Fig. 3c). In fact, the number of such taxonomists involved in monographic revisions targeting the top 20 families was the lowest for the decade of 2010–2019 (Fig. 3c: see the white part of the columns).

In terms of the drivers of taxonomic neglect, the natural logarithm of NI increased significantly with species diversity (the log number of mOTUs detected in our samples; coefficient ± standard error (SE): 0.656 ± 0.152, *t* = 4.318, *P* = 0.0005) and decreased with the logarithmic mean body size of the taxon (coefficient ± SE: −1.102 ± 0.188, *t* = −5.877, *P* = 0.00002). For the expanded dataset, the corresponding numbers for species diversity were coefficient ± SE: 0.721 ± 0.156, *t* = 4.631, *P* = 0.0002 and for body size coefficient ± SE: −1.222 ± 0.203, *t* = −6.029, *P* = 0.00001.

## Discussion

With more than 80% of species undescribed, insects arguably remain the key taxonomic challenge for understanding animal diversity. We here reveal that the same 10–20 clades ranked as families dominate communities of flying insects around the world, when sampled by Malaise traps. This convergence is remarkable, given that the samples were collected across several distinct climatic zones and habitat types, including tropical rainforests, montane forests, cedar savannah, bushwillow woodlands, thorn veld, mangroves and marshes. The biodiversity challenge posed by these ‘dark taxa’ is formidable given their high species diversity and turnover at most sites. A prime example is Cecidomyiidae (gall midges), a globally hyperdiverse taxon that dominates insect communities in terms of species counts and abundance^25^. Yet, regardless of its widespread dominance, this family has received little taxonomic attention.

In striking evidence for the consistency of community composition, we find that two-thirds of the variance across Malaise trap samples is explained by family membership of a species. This raises the question of how such a pattern can emerge across widely different ecosystems and large geographic scales. One explanation could have been that dominant insect families were older, and thus had spread earlier and diverged for a longer time in each part of the world. However, we detected no correlation between species richness per family and clade age (*r* = 0.0039; *n* = 10,023, *P* = 0.70, Supplementary Fig. 5). Another potential explanation for large-scale convergence in community composition might have been widespread species appearing in communities around the world. However, fewer than 3% of species in the top 20 families were found at multiple sites (1–9% in any given family, Supplementary Table 2). Instead, the convergence of taxonomic composition is more likely due to high diversification rates and/or high evolutionary plasticity of taxa ranked above the species and below the family level. Such pronounced adaptability may have allowed these taxa to diversify across habitats and climatic zones. In support of this notion, most species belonging to the top 20 families rely on resources widely available in most habitats (for example, plants, fungi and insect hosts) that are likely to require species-specific adaptations for exploitation^26,27^. This hypothesis should be tested by clade-specific research given that the species diversity within subclades ranked below the family level often varies considerably. For instance, of the ten subfamilies of Cecidomyiidae, one (‘Cecidomyiinae’) contains >70% of described gall midge diversity. Similarly, a single subfamily (Psychodinae) among the seven subfamilies of Psychodidae contains 59% of described drain fly diversity (CoL: https://www.catalogueoflife.org). Similarly, 45% of described diversity of ants (Formicidae) derives from one of its 20 subfamilies (Myrmicinae). The next step in understanding these patterns would be detailed analyses of diversification rates and colonization events for clades below the family and above the species level for the dominant taxa. Such analysis applied to the much less diverse clade comprising snake species identified a few rapidly diversifying lineages, which dominate globally^11^. These clades (for example, Colubrinae) are apparently superior colonizers and ecologically successful in geographically disparate regions regardless of the presence of other taxa. However, compared with most insect clades encountered in our study (for which the mean age is 158 mya), colubrines are very young (with an age of <50 mya), and the patterns here reported for insect have thus evolved over a much greater span of time.

What should be noted is that our study analyses only those insect taxa that are captured in Malaise traps. These traps are particularly effective for sampling flying insects^17,28^ and used widely in large-scale insect biomonitoring programmes^15,16^ even though they are known to mostly target weak fliers active at ground level. The samples thus contain few canopy species, strong fliers and taxa that drop to the ground when encountering an obstacle (for example, many beetles). Such taxa are best sampled with targeted sweep-netting or other traps such as pitfall, leaf-litter, flight intercept, suction or automated light traps^29,30^. It will thus be critical to repeat similar comparative and large-scale analyses of the taxonomic composition of insect communities based on samples obtained with these methods. Such analyses will eventually reveal whether the order-level dominance of Diptera and Hymenoptera^31^ in Malaise trap samples is widespread enough that these orders will surpass the global species richness of Coleoptera and Lepidoptera^32^. In tentative support of such dominance, we note that, among the top 20 families identified in the current study, 10 are dipteran (Cecidomyiidae, Ceratopogonidae, Chironomidae, Psychodidae, Sciaridae, Phoridae, Dolichopodidae, Muscidae, Sphaeroceridae and Chloropidae) and 6 hymenopteran (Platygastridae, Formicidae, Braconidae, Ichneumonidae, Eulophidae and Bethylidae) (Fig. 2a). The diversity of the top taxa observed is exceptionally high by absolute standards, and the species turnover is very high. Thus, we would like to stress that the patterns reported for Malaise traps cannot be attributed to an over*-*representation of taxa although some other taxa may be under-represented.

Overall, the current study is a demonstration of the problems that can be caused by taxonomic impediments. In this case, they are used to interfere with understanding the biodiversity community patterns of flying insects. What allowed us to now address this community structure at the species and family level was a combination of new high-throughput species discovery methods, achieved via large-scale barcoding^33^ and efficient taxonomic assignment techniques. Such technological developments help with community analysis, but also provide partial solutions to overcoming taxonomic impediments. Large-scale barcoding can generate sequence information for large numbers of small insects at a cost of less than 10 cents per specimen in laboratories that require minimal equipment^33^. The process becomes even more efficient when imaging and specimen handling is robotic and/or semiautomatic and the images are used to train convolutional neural networks^34^. In the future, this may ease research on dark taxa by allowing for identifications based on images alone. Efficient barcoding and imaging are essential for implementing a ‘reverse-workflow taxonomy’^8,19,35,36^, where bulk samples are first sorted on the basis of DNA barcodes, before nuclear markers or morphology are used to test whether the clusters delimited by barcodes constitute species. If followed by automated ways to generate species descriptions, it will efficiently help with addressing the species description shortfall for dark taxa.

As biodiversity loss is threatening environmental health globally, obtaining unbiased biodiversity information across all taxa is crucial. Such unbiased information will be important for complementing the vast amount of data already compiled for large and charismatic species^37^. By extension, it will bring the taxa that dominate ecosystems in terms of species diversity, abundance and biomass^38^ into the realm of future biodiversity assessments.

Arguably, one of the most worrying findings of our study is the persistent taxonomic neglect of some of the most important insect families. We found that the more a family contributed to insect communities around the world, the more it has been neglected. Furthermore, we found no evidence for any increase in the taxonomic attention being paid to neglected taxa over time. On the contrary, the neglect proved most severe for the most diverse insect families. It is particularly serious for taxa with small average body size, a pattern that is also well known for beetles where large-bodied species are discovered and described earlier than small species^39,40^. Neglecting species-rich taxa containing many small species thus seems a major shortcoming of modern biodiversity science.

As one among many unfortunate outcomes, the neglect of dominant taxa compromises current estimates of global species richness. These types of estimates frequently use ratios of species richness across families (for example, ratio of butterflies diversity to known insect diversity in Britain^39^) as a basis for extrapolation. If the richness estimates for dark taxa were incorrect, then such estimates would be severely affected. For instance, in the United Kingdom, only 2.7% of described insect diversity belong to Cecidomyiidae^41^, compared with an average of 20% found in the Malaise trap communities analysed here. Assuming that the true diversity of Cecidomyiidae is closer to 20% of the British fauna, then the global species richness estimate would shift from 5.4–7.2 million to 6.5–8.7 million species—even though we are here revising the diversity estimate for only a single dark taxon (Supplemenry Material 1). Thus, the neglect of dark taxa could severely affect our perception of how life on Earth is organized, and there is an urgent need to start intensive work on these taxa to reveal the true species diversity of our planet (‘dark taxon biology’).

Overall, our study suggests that biodiversity research on 10–20 insect families should be a global priority, given the immense gap between our state of knowledge versus the likely importance of these taxa. To understand the functional importance of the key taxa uncovered, similar scalable and new approaches are also needed to reveal the biology of these species including their interactions with other species. Such progress can be achieved through new approaches to taxonomy such as the reverse workflow^19^. They can facilitate the collaboration between taxonomists and molecular ecologists, as the same vouchers can be used to describe species and to gain insights into their biology (for example, by sequencing gut content). Close collaboration of this type will allow for a step change in biodiversity research, conditional on adequate resources being directed to priority taxa.

## Methods

### Datasets, sample collection and processing

The study used DNA barcode generated for full Malaise trap samples across the globe. New datasets were generated for 24 samples from different habitat types in Singapore. Samples were recovered between 3 May 2019 and 9 May 2019 from traps placed at the site for a week before the collection date. All 24 samples were preserved in molecular-grade ethanol before processing all specimens using the high-throughput DNA barcoding pipeline described below. Six of these samples had <100 specimens and were subsequently excluded from analysis. The remaining 18 samples covered a terrestrial forest (5 traps), a mangrove forest (7 traps), coastal forests (3 traps) and a marsh (3 traps). The new data for Singapore were complemented with data from published studies that had sequenced all specimens from a large number of Malaise traps placed in the following countries: Germany^16^, Canada^42,43^, South Africa^44^, Pakistan^45^, Saudi Arabia^45^, Egypt^45^ and Honduras^46^ (Supplementary Table 1). To avoid strong geographic biases, we used only data for 9 of the 20 Malaise traps from South Africa (Kruger National Park), each representing a different habitat. For the study by Telfer et al. (2015) (ref. ^43^), we limited our analysis to the largest sample.

### DNA sequencing, barcoding and identification

Insects from Malaise traps placed in Singapore were processed in a similar approach to Yeo et al. (2021) (ref. ^20^) and Srivathsan et al. (2021) (ref. ^33^) in that we used next-generation sequencing barcoding methods^35^. Briefly, DNA was extracted using 10–30 μl HotSHOT^47^ per specimen and heated to 65 °C for 18 min, followed by 98 °C for 2 min, after which an equal volume of neutralization buffer was added. A 313 bp fragment of *cox1* was amplified using primers mlCO1intF and 5′-GGWACWGGWTGAACWGTWTAYCCYCC-3′ (ref. ^48^) and jgHCO2198: 5′-TANACYTCNGGRTGNCCRAARAAYCA-3′ (ref. ^49^). The primers were tagged with a 13 bp tag at the 5′ end designed for MinION-based barcoding^18,33^ and 9 bp tags for Illumina-based barcoding^19^ (Supplementary Data 1). Polymerase chain reactions (PCRs) were conducted in 96*-*well plates using one negative control per plate, and each PCR mix contained 8 μl Mastermix (CWBio), 0.5 μl bovine serum albumin (1 mg ml^−1^), 0.5 μl MgCl_2_ (25 mM), 1 μl each of primer (10 μM) and 4–7 μl of template DNA. The cycling conditions were: 5 min initial denaturation at 94 °C followed by 35 cycles of denaturation at 94 °C (30 s), annealing at 45 °C (1 min) and extension at 72 °C (1 min), followed by final extension of 72 °C (5 min). A subset of 8–15 products per plate were run in agarose gels to assess PCR success. Samples were pooled and purified using Ampure XP beads (Beckman Coulter). Pooled samples were sequenced either using Illumina HiSeq 2500 (2 × 250 bp) or MinION (Oxford Nanopore Technologies). Illumina sequencing was outsourced while MinION sequencing was conducted in-house using an R9.4 flowcell. Libraries were prepared using the SQK-LSK109 Ligation Sequencing Kit with two recommended modifications^33^. Firstly, the end-repair reaction consisted of 50 μl of DNA in molecular-grade water, 7 μl of Ultra II End-prep reaction buffer (New England Biolabs) and 3 μl of Ultra II End Prep enzyme mix (New England Biolabs). Secondly all clean-ups using Ampure beads were conducted at 1× ratio. Fast basecalling model as implemented in Guppy was used as high-accuracy basecalling was not available at the time of data processing. The 1D MinION reads that have estimated raw accuracy ~90% (ref. ^50^) were then converted into DNA barcodes using error corrections that have been shown to yield DNA barcodes that are virtually identical to barcodes obtained with Sanger or Illumina sequencing (99.99% accuracy^18^).

Data analysis of the Illumina reads started with paired-end read merging using PEAR^51^. Reads were demultiplexed allowing for up to a 2 bp mismatch in primer sequences, while no mismatch was allowed in the tag sequence. Demultiplexed reads for each specimen were merged to form unique sequences, and only amplicons having at least 50 sequences were processed further. A dominant sequence was identified, and if it had a read count exceeding 10, it was ‘called’ as the DNA barcode for the specimen, as long as it was also at least five times as common as the second dominant sequence. MinION sequence data were processed using minibarcoder^18,52^, which both demultiplexes the data and calls the barcodes. The final consolidated barcode sets were used for further analysis.

Barcodes were clustered at 1% using objective clustering (see below), and specimens were sorted physically on the basis of their cluster assignments. For Singapore samples, each cluster was morphologically identified to family. For Lepidoptera specimens, as well as for a small number of other specimens where morphological identification was not possible, we assigned specimens to families on the basis of DNA characters alone. This was done by conducting BLAST against NCBI-nt database as well as searches against the BOLD Systems Identification engine (https://boldsystems.org/index.php/IDS_OpenIdEngine). A taxonomic assignment to family was accepted if there were no conflicting family-level matches in the top 20 unique matches. For all other morphologically identified specimens, DNA-based identification was examined and any conflict with morphology was resolved through re-examination of morphology. If a conflict persisted, the specimen was not identified to family. Taxonomic classifications for published studies were based on metadata provided by the studies. These studies employed various methods of identification including morphology, matches on BOLD, and tree-based identifications. It was noted that several Hymenoptera identified on the basis of morphology as Scelionidae matched Platygastridae on BOLD Systems. This is probably due to recent classification changes. The ‘old’ Platygastridae (before 2007) was treated only as Platygastroidea (Platygastridae = Scelionidae) until very recently when it has been split into several families^53^. We here follow several other recent studies^43,45^ that have used the old circumscription of Platygastridae.

### Species delimitation

Before species delimitation, we excluded sequences that contained a stop codon when translated using the invertebrate mitochondrial genetic code. Secondly, to ensure that the large datasets had sufficient sequence overlap for multiple sequence alignments, short barcodes were excluded. Any sample/trap that contained <100 barcode sequences was excluded. For datasets containing 313 bp barcode sequences, the length cut-off was 300 bp, while the cut-off was 500 bp for datasets containing 658 bp barcodes. Barcodes were aligned using MAFFT v7 (ref. ^54^). Species delimitation was conducted using objective clustering, which is a distance-based clustering algorithm originally described in Meier et al. (2006) (ref. ^23^). Species delimitations were also conducted using another distance-based approach Assemble Species by Automatic Partitioning (ASAP)^24^ and a tree-based approach (Poisson Tree Processes or PTP)^55^. For PTP-based species delimitation, phylogeny was constructed using RaxML (v8.2.5) and species delimitation was conducted using mPTP (v 0.2.4,–single,–ml). Most analyses initially used mOTUs obtained with objective clustering using a 3% distance threshold before testing the results with ASAP and PTP. We find that the results are very similar irrespective of clustering method or distance threshold (Supplementary Table 1). Species delimitations were performed for individual datasets independently. An estimate for total species diversity was obtained using USEARCH (v 11.0.667) (ref. ^56^) cluster_fast (-sort length -id 0.97) for the 225,261 sequences used in the study.

### Statistical analyses of community composition

All analyses were conducted in R v4.1.2 (ref. ^57^). Analyses were limited to insects (that is, spiders, Collembola and so on were excluded: see list in Supplementary Data 2: Tables 14 and 15) and species that could be identified to family (leading to exclusion of 0.05–11.6% of the mOTUs, Supplementary Table 1). Overall sequences from 225,261 specimens were analysed. Two different datasets were studied: one where all sequences available for the same Malaise trap were combined (39 traps, main dataset) and one where the sample-level resolution for the datasets from Germany and Canada was retained (56 datasets, expanded dataset). This was feasible due to availability of high-quality metadata such that weekly samples could be treated separately. Community composition at the family level was analysed using a linear model. Here we first logarithmically transformed the proportion of mOTUs for each dataset, adding 0.01 to zero proportions (since (log(0) is undefined). We then modelled the transformed response variable as a function of family [lm(log(Proportion + 0.01)~Taxon,data = dataset)], using adjusted *R*^2^ values as the key statistic of variance explained. Furthermore, we ran a PCA on the community matrix of each site (with cell values equalling the proportion of species richness per family) using rda. We set scale as FALSE, and used a barplot of relative eigenvalues to assess the percentage variation explained by each principal component.

The top 20 families were identified on the basis of ranking of average proportion of mOTUs per family. To test whether the subjective nature of family ranks influence the results, we also examined which taxa were in the top 21–30 taxa. We then merged each with its sister clade on the basis of recent phylogenies, to test whether the merge would generate a taxon that would be included in the list of top 20 families. Next, we examined whether the high number of species in these clades across Malaise trap samples was due to high species-level dispersal rates. We analysed species turnover across ‘sites’. ‘Sites’ were broadly defined as Canada, Egypt, Germany, Honduras, Saudi Arabia, Pakistan, South Africa and Singapore.

Lastly, to assess whether taxonomic dominance (in terms of species richness at the family level) could be attributed to the evolutionary age of the taxon, we examined in the correlation between the age of family and the proportion of mOTUs. Family ages were obtained from TimeTree (http://timetree.org, beta version 5), with missing values obtained from major large-scale studies involving dating^58–62^. For the various statistical analyses, we excluded families with ≤10 specimens across all the samples and families that are present in one sample only.

### Assessment of taxonomic neglect

To assess how much taxonomic attention has been given to the families dominating Malaise trap samples, the total number of species described for each of the top 20 families was obtained from CoL v22.3 (https://www.catalogueoflife.org/). For Bethylidae, CoL lacked information although the family was listed. We thus used values from a recent checklist instead^63^. For two families, we used the species richness in superfamilies (Noctuoidea and Platygastroidea). This was either because the family was not listed (Erebidae) or because of recent changes in family-level classification (Platygastridae, see above).

To next characterize the level of taxonomic neglect, we defined an NI as *N*_mOTU_/*N*_sp_, where *N*_mOTU_ is the number of mOTUs for a given family across the whole dataset and *N*_sp_ is the total number of species described as obtained from CoL. To evaluate potential drivers of taxonomic neglect, we next hypothesized that small-bodied or species-rich insect groups would be particularly prone to neglect, as being inconspicuous, poor in morphological characters and phylogenetically and/or taxonomically unwieldy (as due to their diversity alone). Large-bodied or species-poor families, we predicted, would be considered charismatic, accessible to morphological assessment and phylogenetically and/or taxonomically more clear cut. To test for such impacts, we modelled ln(NI*)* as a function of the mean body size of the insect family and the diversity of OTUs. To obtain diversity of OTUs, for each of the top 18 families and 2 superfamilies, species delimitation was conducted independently using objective clustering. The body range size limits for the calculation of mean-of-logs body-size was obtained from Rainford et al.^64^. For Crambidae, we used body-size range of Pyralidae, given that the study included Crambidae within Pyralidae. Similarly for Aleyrodidae, we used the body-size range for Aleyrodoidea. For Erebidae, the minimum and maximum forewing length was based on the combination of Lymantriidae and Arctiidae. For Platygastridae, it was based on combination of Platygastridae and Scelionidae. We modelled the relationship between neglect, species diversity and body size as ln(NI) ~ ln(*N*_mOTU_) × mean-of-logs body-size. Since we detected no interaction between ln(*N*_mOTU_) and mean-of-logs body-size (main dataset: coefficient ± SE: = −0.305 ± 0.227, *t* = −1.342, *P* = 0.198; expanded dataset: coefficient ± SE: = −0.141 ± 0.434, *t* = −0.324, *P* = 0.75), this term was removed from the final model (ln(NI) ~ ln(*N*_mOTU_) + mean-of-logs body-size).

We next examined the taxonomic attention given to the top 20 taxa over time. To this aim, we counted the number of species descriptions in Zoological Record. All data for the top 20 families were downloaded by search term ST = [Taxon name], as encompassing 181,985 studies. Studies in the past four decades (1980–2019) that describe species were identified by the sp nov epithet in the organism field. This approach identified 16,362 studies. The information on organism was then extracted to obtain the species and the family name, along with information on the year of publication and the authors involved. The data were then parsed to obtain the total number of species described in the study. For Erebidae, Crambidae, Aleyrodidae and Platygastridae, we assessed information at the level of the superfamily.

To test for a change in the relation between species diversity and neglect over time, we tested for an interaction between NI and Decade. To this aim, we compared two analysis of covariance models fitted to the univariate data: lm(log(*N*_sp10_) ~ log(NI), data = UnivariateData), and lm(formula = log(*N*_sp10_) ~ log(NI) × Decade, data = UnivariateData). Here, *N*_sp10_ is the number of species described in a decade, with decades being 1980–1989, 1990–1999, 2000–2009 and 2010–2019. The fit of the two respective models was then compared by ANOVA (anova (model1,model2)).

Lastly, to further evaluate whether taxonomic work dedicated to the top 20 families has changed over time, we extracted information on the number of authors highly dedicated to a particular family, as scored from the number of authors exceeding a particular threshold (*S*_threshold_) of species descriptions. Given that some of these descriptions involved multiple authors, for each author *i*, the score was calculated as *S*_*i*_ = ${\sum} {\frac{1}{{N_{{\mathrm{auth}}\_j}}}}$, where $N_{{\mathrm{auth}}\_j}$ is the number of authors in the study *j*. For an article in which two authors described a species, each author would thus get an author score of 0.5 for this species. The number of highly dedicated authors was then scored as the number of authors with S_*i*_ > *S*_threshold_ per decade, where *S*_threshold_ = 50.

### Reporting summary

Further information on research design is available in the Nature Portfolio Reporting Summary linked to this article.

## Supplementary information

Supplementary Information Supplementary Figs. 1–5, Tables 1–3 and information on ‘Estimation of true global insect species diversity’.

Reporting Summary

Supplementary Data 1 Primer tags for barcoding.

Supplementary Data 2 Species delimitation data for samples, Supplementary Tables 4–15.

## Data Availability

Summarized data have been included as part of Supplementary Information. Barcode datasets have been uploaded to figshare (10.6084/m9.figshare.20449401)^65^, and data from Singapore identified to family have been submitted to GenBank (accession numbers: OQ476881–OQ503166).

## Code availability

Scripts to summarize the results are available at GitHub: https://github.com/asrivathsan/malaisetraps.

## References

[1] Summary for policymakers of the global assessment report on biodiversity and ecosystem services of the Intergovernmental Science-Policy Platform on Biodiversity and Ecosystem Services. *Intergovernmental Science-Policy Platform on Biodiversity and Ecosystem Services* (eds Díaz, S. et al.) (IPBES Secretariat, 2019); 10.5281/zenodo.3553579

[2] The Global Risks Report 2020. *World Economic Forum* https://www.weforum.org/reports/the-global-risks-report-2020 (2020).

[3] An eco-wakening: Measuring global awareness, engagement and action for nature. *Economist Intelligence Unit*; https://impact.economist.com/sustainability/ecosystems-resources/an-eco-wakening-measuring-global-awareness-engagement-and-action-for-nature (2021).

[4] Rohr JR, Mahan CG, Kim KC (2007). Developing a monitoring program for invertebrates: guidelines and a case study. Conserv. Biol..

[5] Wilson EO (2017). Biodiversity research requires more boots on the ground. Nat. Ecol. Evol..

[6] Mora C, Tittensor DP, Adl S, Simpson AGB, Worm B (2011). How many species are there on Earth and in the ocean?. PLoS Biol..

[7] Barrowclough GF, Cracraft J, Klicka J, Zink RM (2016). How many kinds of birds are there and why does it matter?. PLoS ONE.

[8] Hartop E, Srivathsan A, Ronquist F, Meier R (2021). Towards large-scale integrative taxonomy (LIT): resolving the data conundrum for dark taxa. Syst. Biol..

[9] Wiens JJ (2007). Global patterns of diversification and species richness in amphibians. Am. Nat..

[10] Ricklefs RE, Renner SS (2012). Global correlations in tropical tree species richness and abundance reject neutrality. Science.

[11] Wiens JJ (2018). Patterns of local community composition are linked to large-scale diversification and dispersal of clades. Am. Nat..

[12] Losey JE, Vaughan M (2006). The economic value of ecological services provided by insects. BioScience.

[13] Outhwaite CL, McCann P, Newbold T (2022). Agriculture and climate change are reshaping insect biodiversity worldwide. Nature.

[14] van Klink R (2020). Meta-analysis reveals declines in terrestrial but increases in freshwater insect abundances. Science.

[15] Karlsson D, Hartop E, Forshage M, Jaschhof M, Ronquist F (2020). The Swedish Malaise Trap Project: a 15 year retrospective on a countrywide insect inventory. Biodivers. Data J..

[16] Geiger MF (2016). Testing the Global Malaise Trap Program– how well does the current barcode reference library identify flying insects in Germany?. Biodivers. Data J..

[17] Noyes JS (1989). The diversity of Hymenoptera in the tropics with special reference to Parasitica in Sulawesi. Ecol. Entomol..

[18] Srivathsan A (2019). Rapid, large-scale species discovery in hyperdiverse taxa using 1D MinION sequencing. BMC Biol..

[19] Wang WY, Srivathsan A, Foo M, Yamane SK, Meier R (2018). Sorting specimen-rich invertebrate samples with cost-effective NGS barcodes: validating a reverse workflow for specimen processing. Mol. Ecol. Resour..

[20] Yeo D (2021). Mangroves are an overlooked hotspot of insect diversity despite low plant diversity. BMC Biol..

[21] Montgomery GA, Belitz MW, Guralnick RP, Tingley MW (2021). Standards and best practices for monitoring and benchmarking insects. Front. Ecol. Evol..

[22] Hebert PDN, Cywinska A, Ball SL, deWaard JR (2003). Biological identifications through DNA barcodes. Proc. R. Soc. Lond. Ser. B.

[23] Meier R, Shiyang K, Vaidya G, Ng PKL (2006). DNA barcoding and taxonomy in Diptera: a tale of high intraspecific variability and low identification success. Syst. Biol..

[24] Puillandre N, Brouillet S, Achaz G (2021). ASAP: assemble species by automatic partitioning. Mol. Ecol. Resour..

[25] Hebert PDN (2015). Counting animal species with DNA barcodes: Canadian insects. Philos. Trans. R. Soc. B.

[26] Ehrlich PR, Raven PH (1964). Butterflies and plants: a study in coevolution. Evolution.

[27] Strong, D. R., Lawton, J. H. & Southwood, S. R. *Insects on Plants. Community Patterns and Mechanisms* (Harvard Univ. Press, 1984).

[28] Skvarla MJ, Larson JL, Fisher JR, Dowling APG (2021). A review of terrestrial and canopy Malaise traps. Ann. Entomol. Soc. Am..

[29] Erwin TL (1982). Tropical forests: their richness in Coleoptera and other arthropod species. Coleopterists Bull..

[30] Noyes JS (2012). An inordinate fondness of beetles, but seemingly even more fond of microhymenoptera!. Hamuli.

[31] Kitching RL, Li D, Stork NE (2001). Assessing biodiversity ‘sampling packages’: how similar are arthropod assemblages indifferent tropical rainforests?. Biodivers. Conserv..

[32] Forbes AA, Bagley RK, Beer MA, Hippee AC, Widmayer HA (2018). Quantifying the unquantifiable: why Hymenoptera, not Coleoptera, is the most speciose animal order. BMC Ecol..

[33] Srivathsan A (2021). ONTbarcoder and MinION barcodes aid biodiversity discovery and identification by everyone, for everyone. BMC Biol..

[34] Wührl L (2021). DiversityScanner: robotic handling of small invertebrates with machine learning methods. Mol. Ecol. Resour..

[35] Meier R, Wong W, Srivathsan A, Foo M (2016). $1 DNA barcodes for reconstructing complex phenomes and finding rare species in specimen‐rich samples. Cladistics.

[36] Riedel A, Sagata K, Suhardjono YR, Tänzler R, Balke M (2013). Integrative taxonomy on the fast track—towards ore sustainability in biodiversity research. Front. Zool..

[37] Titley MA, Snaddon JL, Turner EC (2017). Scientific research on animal biodiversity is systematically biased towards vertebrates and temperate regions. PLoS ONE.

[38] Bar-On YM, Phillips R, Milo R (2018). The biomass distribution on Earth. Proc. Natl Acad. Sci. USA.

[39] Gaston KJ (1991). Body size and probability of description: the beetle fauna of Britain. Ecol. Entomol..

[40] Stork NE, McBroom J, Gely C, Hamilton AJ (2015). New approaches narrow global species estimates for beetles, insects, and terrestrial arthropods. Proc. Natl Acad. Sci..

[41] Barnard, P. C. *The Royal Entomological Society Book of British Insects*. (Wiley, 2011). 10.1002/9781444344981

[42] DeWaard JR (2019). Expedited assessment of terrestrial arthropod diversity by coupling Malaise traps with DNA barcoding. Genome.

[43] Telfer AC (2015). Biodiversity inventories in high gear: DNA barcoding facilitates a rapid biotic survey of a temperate nature reserve. Biodivers. Data J..

[44] D’Souza ML (2021). Biodiversity baselines: tracking insects in Kruger National Park with DNA barcodes. Biol. Conserv..

[45] Ashfaq M, Akhtar S, Rafi MA, Mansoor S, Hebert PDN (2017). Mapping global biodiversity connections with DNA barcodes: Lepidoptera of Pakistan. PLoS ONE.

[46] D’Souza ML, Hebert PDN (2018). Stable baselines of temporal turnover underlie high beta diversity in tropical arthropod communities. Mol. Ecol..

[47] Truett GE (2000). Preparation of PCR-quality mouse genomic DNA with hot sodium hydroxide and Tris (HotSHOT). Biotechniques.

[48] Leray M (2013). A new versatile primer set targeting a short fragment of the mitochondrial COI region for metabarcoding metazoan diversity: application for characterizing coral reef fish gut contents. Front. Zool..

[49] Geller J, Meyer C, Parker M, Hawk H (2013). Redesign of PCR primers for mitochondrial cytochrome c oxidase subunit I for marine invertebrates and application in all-taxa biotic surveys. Mol. Ecol. Resour..

[50] Silvestre-Ryan J, Holmes I (2021). Pair consensus decoding improves accuracy of neural network basecallers for nanopore sequencing. Genome Biol..

[51] Zhang J, Kobert K, Flouri T, Stamatakis A (2014). PEAR: a fast and accurate Illumina Paired-End reAd mergeR. Bioinformatics.

[52] Srivathsan A (2018). A MinION^TM^-based pipeline for fast and cost-effective DNA barcoding. Mol. Ecol. Resour..

[53] Chen H (2021). An integrated phylogenetic reassessment of the parasitoid superfamily Platygastroidea (Hymenoptera: Proctotrupomorpha) results in a revised familial classification. Syst. Entomol..

[54] Katoh K, Standley DM (2013). MAFFT Multiple Sequence Alignment Software Version 7: improvements in performance and usability. Mol. Biol. Evol..

[55] Zhang J, Kapli P, Pavlidis P, Stamatakis A (2013). A general species delimitation method with applications to phylogenetic placements. Bioinformatics.

[56] Edgar RC (2010). Search and clustering orders of magnitude faster than BLAST. Bioinformatics.

[57] R: a language and environment for statistical computing (R Core Team, 2021).

[58] Johnson KP (2018). Phylogenomics and the evolution of hemipteroid insects. Proc. Natl Acad. Sci. USA.

[59] Peters RS (2018). Transcriptome sequence-based phylogeny of chalcidoid wasps (Hymenoptera: Chalcidoidea) reveals a history of rapid radiations, convergence, and evolutionary success. Mol. Phylogenet. Evol..

[60] Peters RS (2017). Evolutionary history of the Hymenoptera. Curr. Biol..

[61] Kawahara AY (2019). Phylogenomics reveals the evolutionary timing and pattern of butterflies and moths. Proc. Natl Acad. Sci. USA.

[62] Wiegmann BM (2011). Episodic radiations in the fly tree of life. Proc. Natl Acad. Sci. USA.

[63] Azevedo C (2018). Global guide of the flat wasps (Hymenoptera, Bethylidae). Zootaxa.

[64] Rainford JL, Hofreiter M, Mayhew PJ (2016). Phylogenetic analyses suggest that diversification and body size evolution are independent in insects. BMC Ecol. Evol..

[65] Srivathsan, A. et al. Convergence of dominance and neglect in flying insect diversity. *figshare*10.6084/m9.figshare.20449401 (2022). 10.1038/s41559-023-02066-0 PMC10333119 37202502

