# Supplementary material for: Convergence of dominance and neglect in flying insect diversity
Source: Nat Ecol Evol. 2023 May 18;7(7):1012–21. doi: 10.1038/s41559-023-02066-0 (PMC10333119; doi:10.1038/s41559-023-02066-0)
Supplement: Supplementary file 1 — Supplementary Figs. 1–5, Tables 1–3 and information on ‘Estimation of true global insect species diversity’. [file 41559_2023_2066_MOESM1_ESM.pdf]

nature ecology & evolution

Article https://doi.org/10.1038/s41559-023-02066-0

Convergence of dominance and neglect in flying insect diversity

In the format provided by the authors and unedited

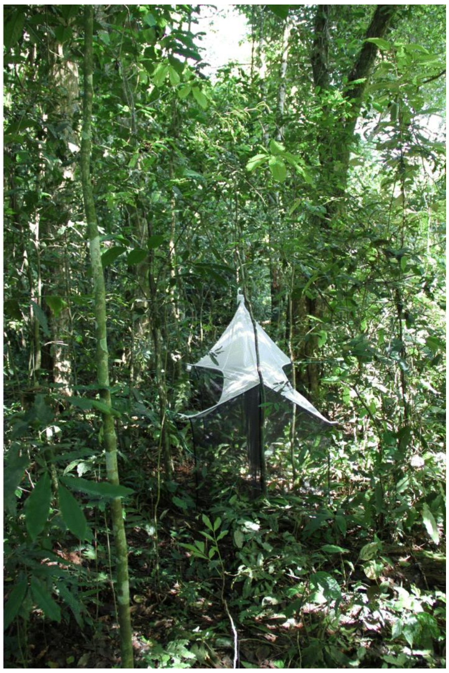

Supplementary Fig 1. A Townes-type Malaise trap

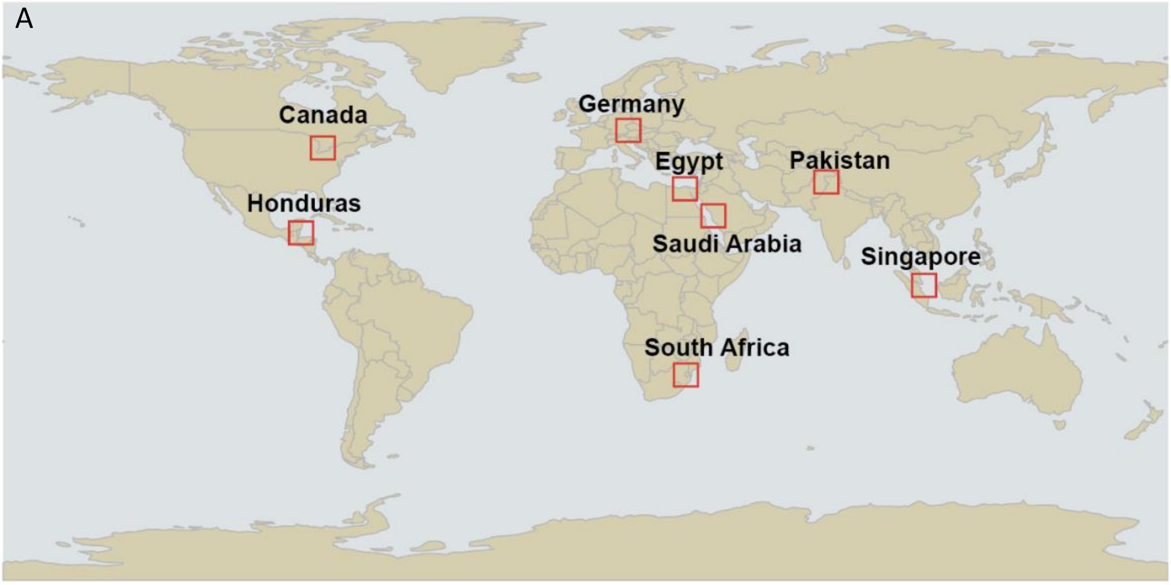

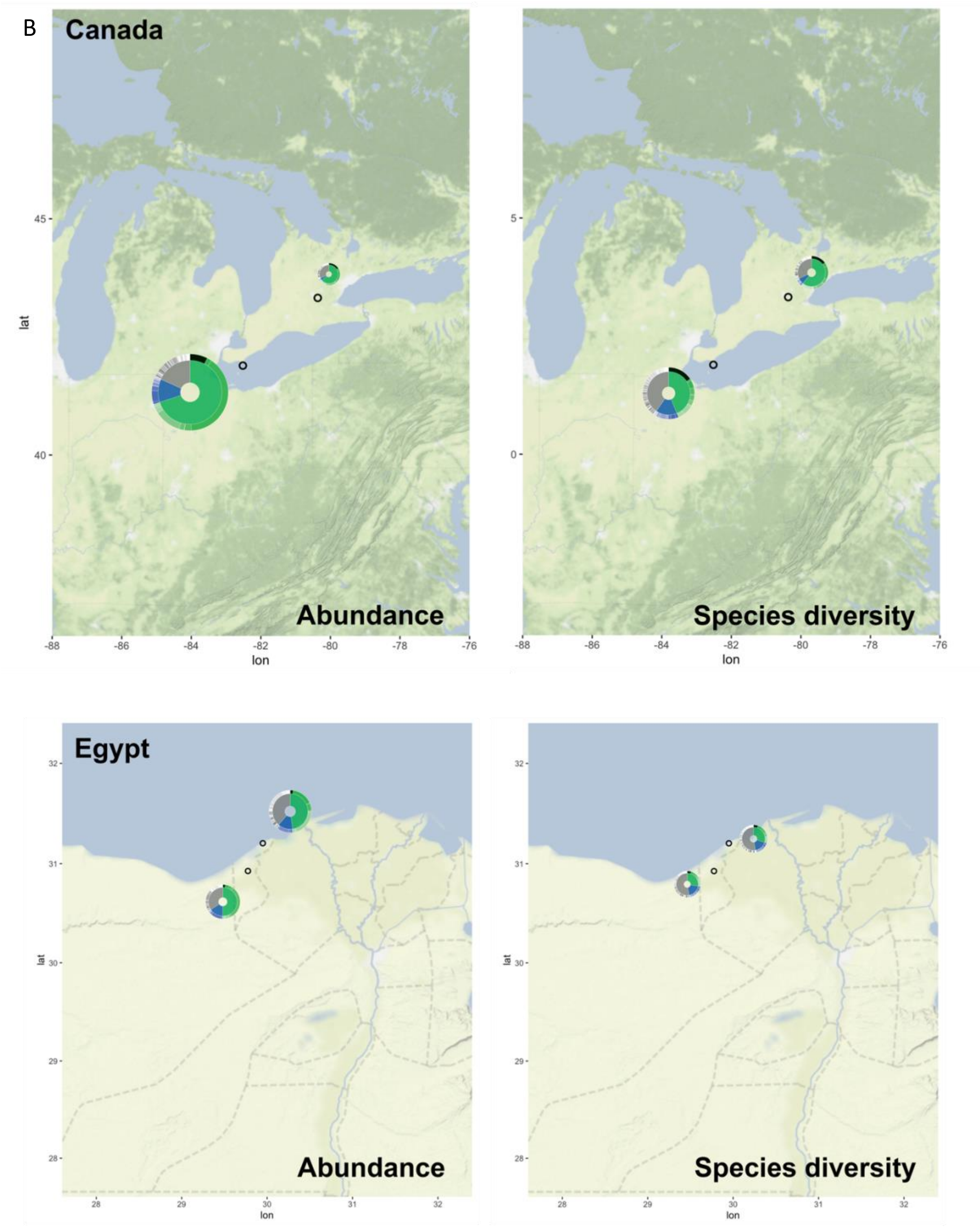

B
Canada
Abundance
Species diversity
lat
45
40
5
0
lon
-88
-86
-84
-82
-80
-78
-76
Egypt
Abundance
Species diversity
lat
32
31
30
29
28
lon
28
29
30
31
32

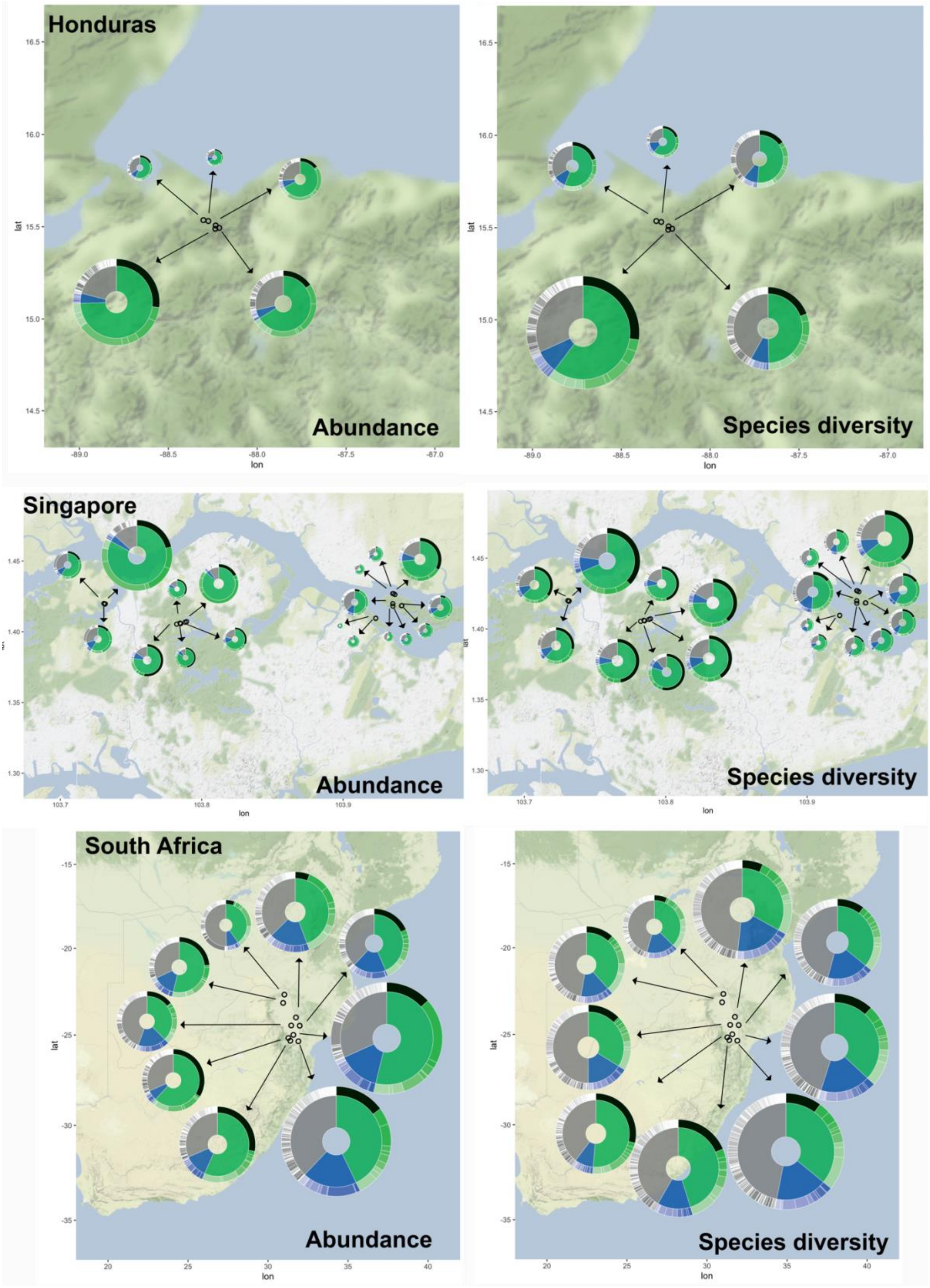

Honduras
lat
lon
Abundance
Species diversity
Singapore
lon
Abundance
Species diversity
South Africa
lat
lon
Abundance
Species diversity

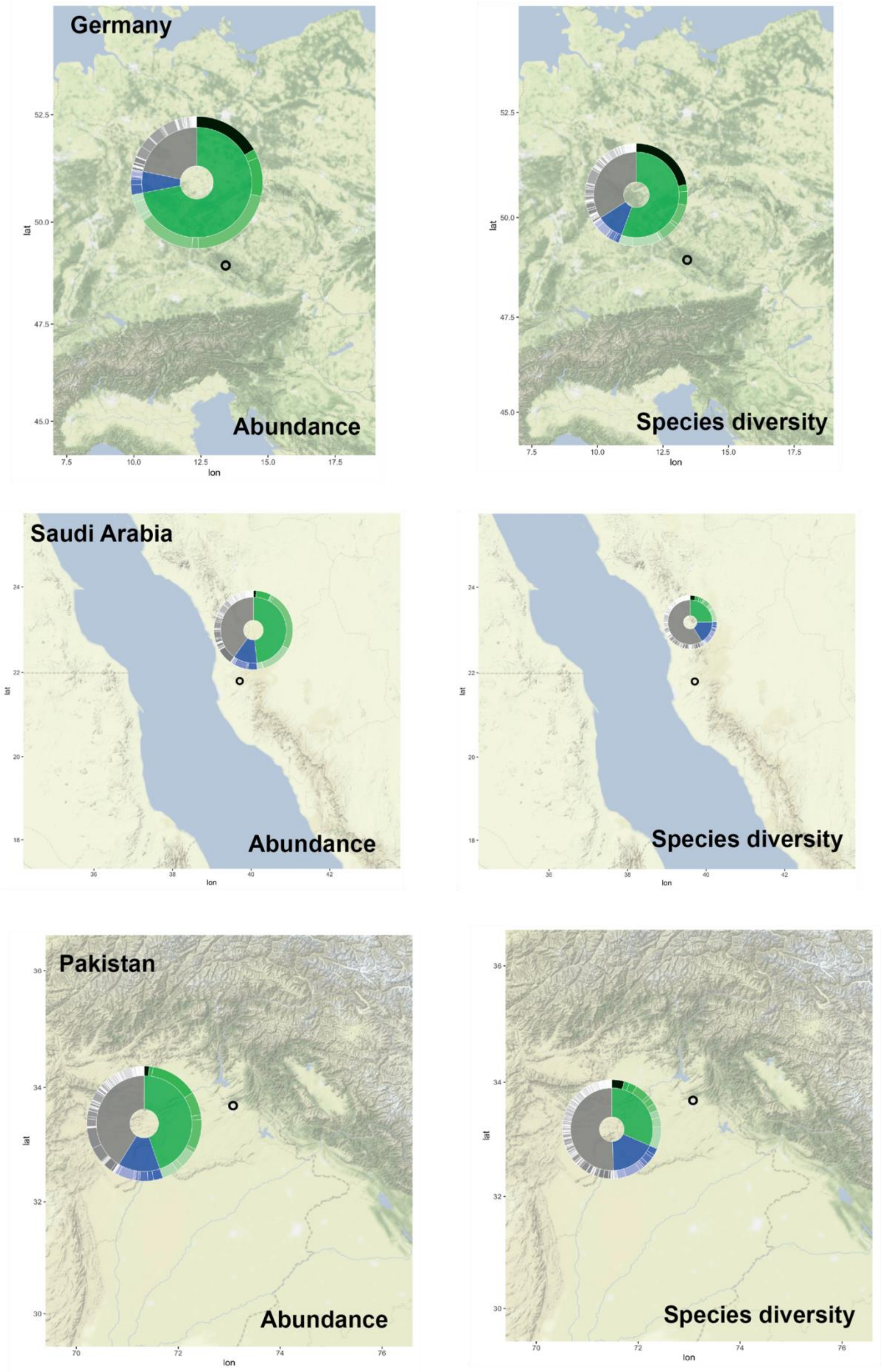
Germany
Abundance
Species diversity
Saudi Arabia
Abundance
Species diversity
Pakistan
Abundance
Species diversity
lat
lon

Supplementary Figure 2: A: Map of sampling sites B: Compositions of Malaise trap samples in terms of abundance (left-hand panels) and species diversity (right-hand panels with precise geolocations for each site. Each chart shows the taxonomic composition of a sample obtained by an individual Malaise trap at a specific site. The inner circle illustrates the proportion of biodiversity in the top 10 (green), next 10 (blue) and remaining families (grey). The outer ring shows what proportion of biodiversity belongs to the top 10, next 10 and the remaining families. Dark green is used to illustrate the extraordinary diversity of Cecidomyiidae (Diptera). All charts are scaled relative to total number of specimens (left-hand panels) and species (right-hand panels) at each site. Map plots were created using ggmap and tiles by Stamen Design (CC-BY-3.0) with data from OpenStreetMap contributors: https://www.openstreetmap.org/copyright.

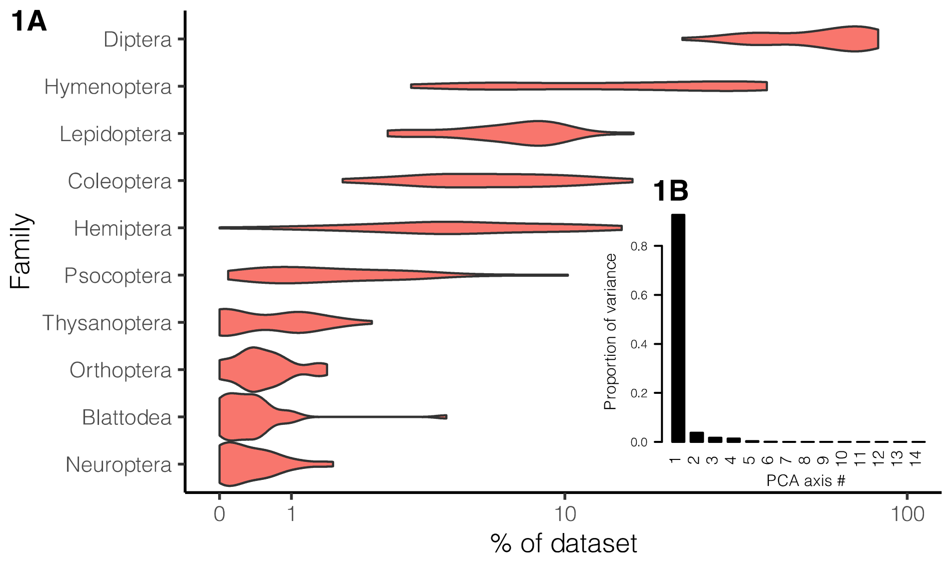
1A
Diptera
Hymenoptera
Lepidoptera
Coleoptera
Hemiptera
Psocoptera
Thysanoptera
Orthoptera
Blattodea
Neuroptera
Family
0
1
10
100
% of dataset
1B
Proportion of variance
0.0
0.2
0.4
0.6
0.8
1 2 3 4 5 6 7 8 9 10 11 12 13 14
PCA axis #

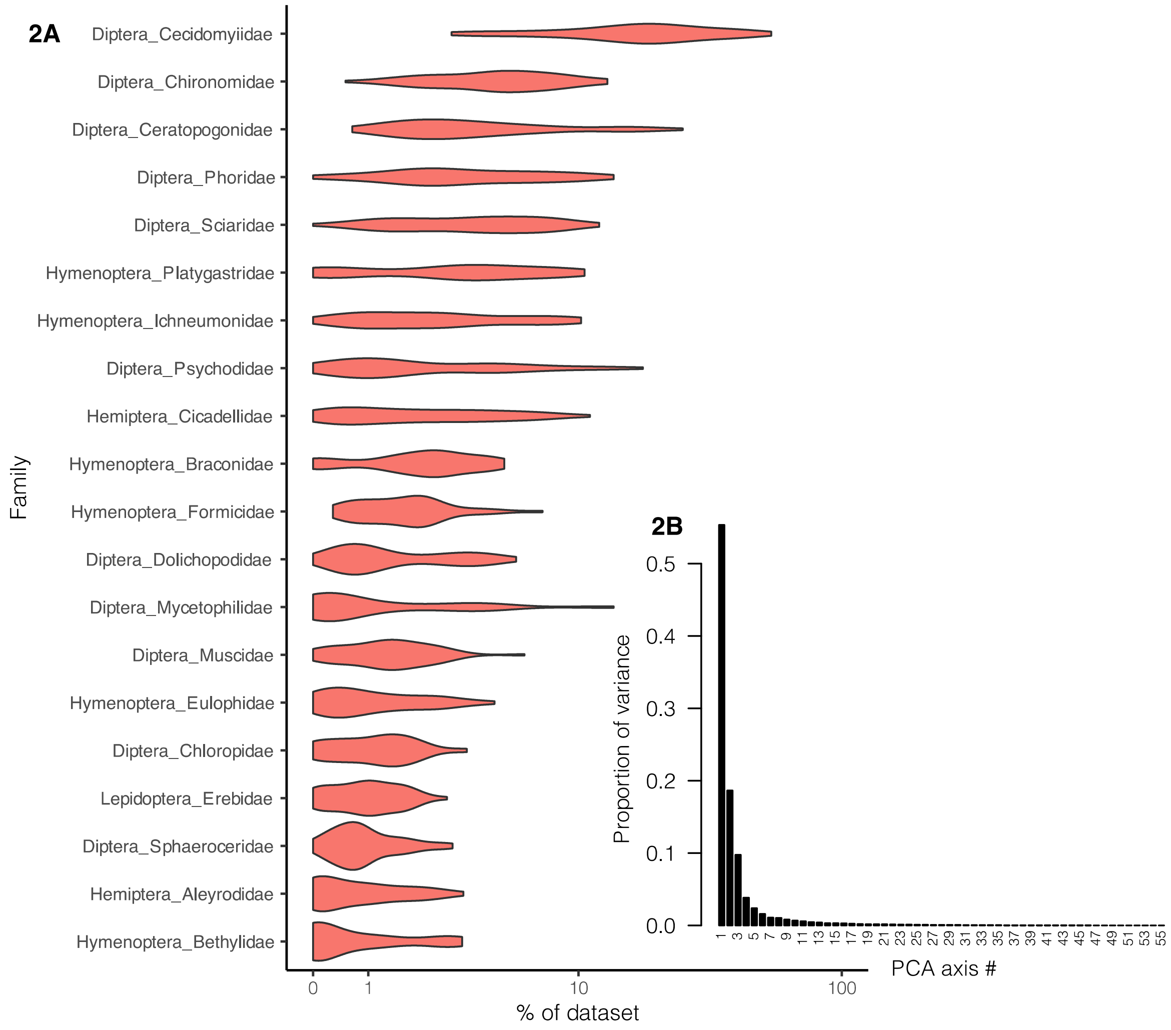
2A
Diptera_Cecidomyiidae
Diptera_Chironomidae
Diptera_Ceratopogonidae
Diptera_Phoridae
Diptera_Sciaridae
Hymenoptera_Platygastridae
Hymenoptera_Ichneumonidae
Diptera_Psychodidae
Hemiptera_Cicadellidae
Hymenoptera_Braconidae
Hymenoptera_Formicidae
Diptera_Dolichopodidae
Diptera_Mycetophilidae
Diptera_Muscidae
Hymenoptera_Eulophidae
Diptera_Chloropidae
Lepidoptera_Erebidae
Diptera_Sphaeroceridae
Hemiptera_Aleyrodidae
Hymenoptera_Bethylidae
Family
0
1
10
100
% of dataset
2B
Proportion of variance
0.0
0.1
0.2
0.3
0.4
0.5
PCA axis #

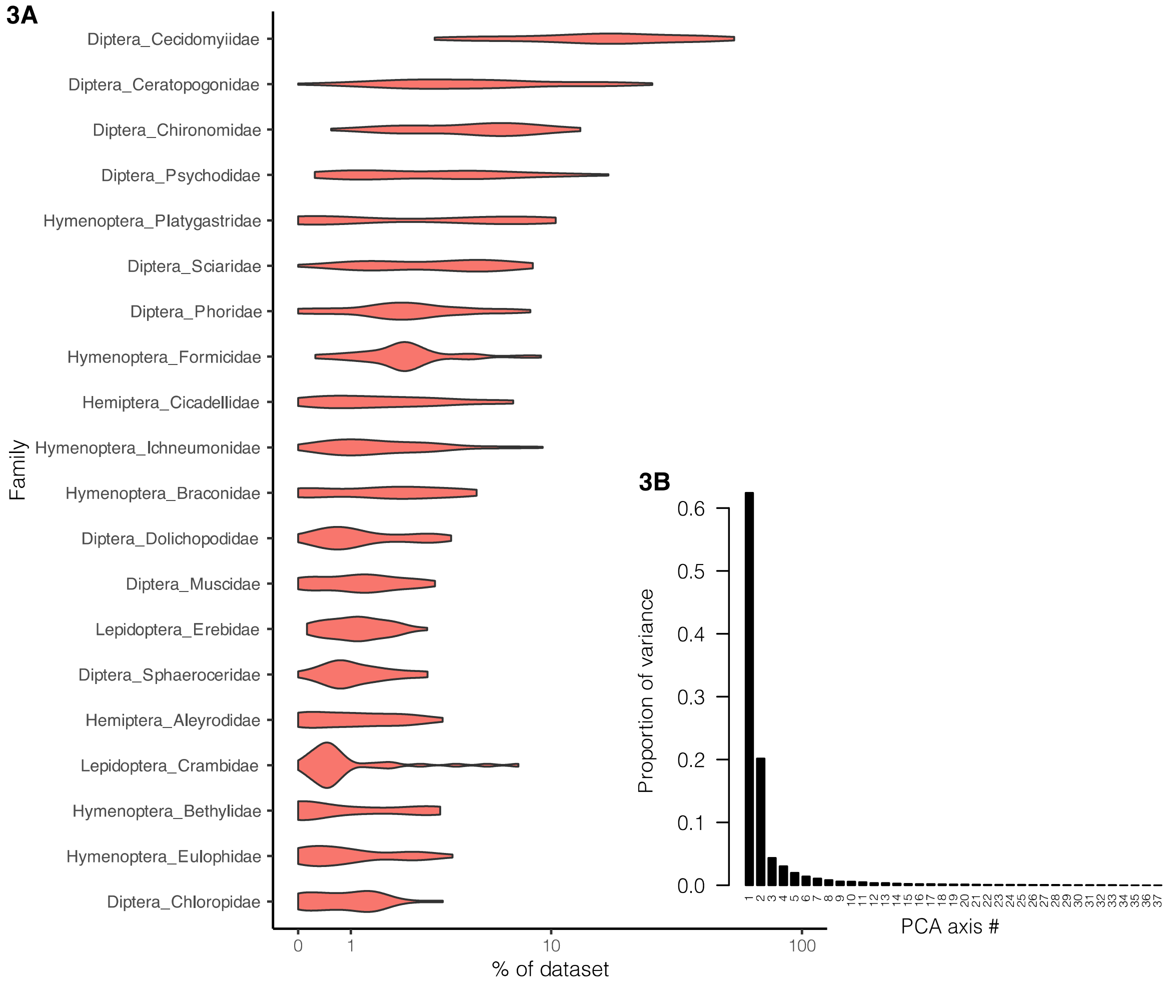

Supplementary Fig 3. Consistency in community composition of insects in terms of 1: insect orders, species composition determined by objective clustering 3%, main dataset, 2: insect families, species composition determined by objective clustering 3%, expanded dataset and 3: insect families, species composition determined by ASAP, main dataset. (A) proportional (%) species richness of all insect orders (panel 1), or the top 20 families among sites (x-axis log-transformed) (panel 2 and 3) and (B) proportion of variance absorbed by the first axis in a principal component analysis of variation in the proportion of species richness per insect order/family. Algorithm failed for species delimitation using ASAP for TrapW from dataset from South Africa, and this trap was excluded for the analysis corresponding to panel 3. These figures show the results of analyses done in addition to the analysis presented in the main manuscript Figure 2, which is based on insect families, species composition determined by objective clustering 3% and main dataset.

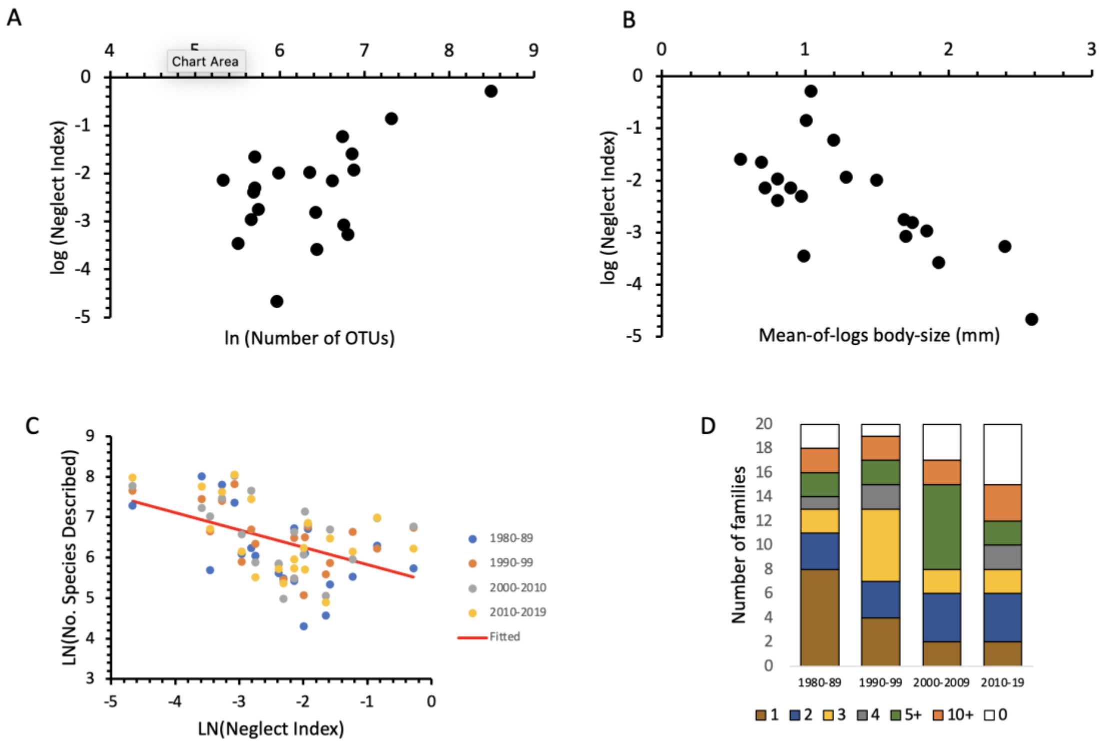

Supplementary Fig 4. Taxonomic neglect species diversity, and taxonomic activity dedicated to the top-20 families. This figure shows results for the expanded data set, whereas Fig. 3 in the main text shows results for the main data set (for a distinction between these sets, see Methods). (A) Taxonomic neglect (as expressed by the Neglect Index, NI) increases with the species diversity of the target taxon, and (B) taxonomic neglect decreases with increasing body size of the target taxon. (C) The more neglected a taxon is, the less taxonomic attention is dedicated to it – with no sign of improvement over time. (D) Likewise, the number of authors publishing monographic work on the top-20 families shows no increase over time. The stacked bars show the proportion of families with 0 (white),1 (brown), 2 (blue), 3 (yellow), 4 (grey), 5-9 (green) and 10+ (orange) authors having described >=50 species in a decade. See Figure 3 of main manuscript for corresponding results of main dataset.

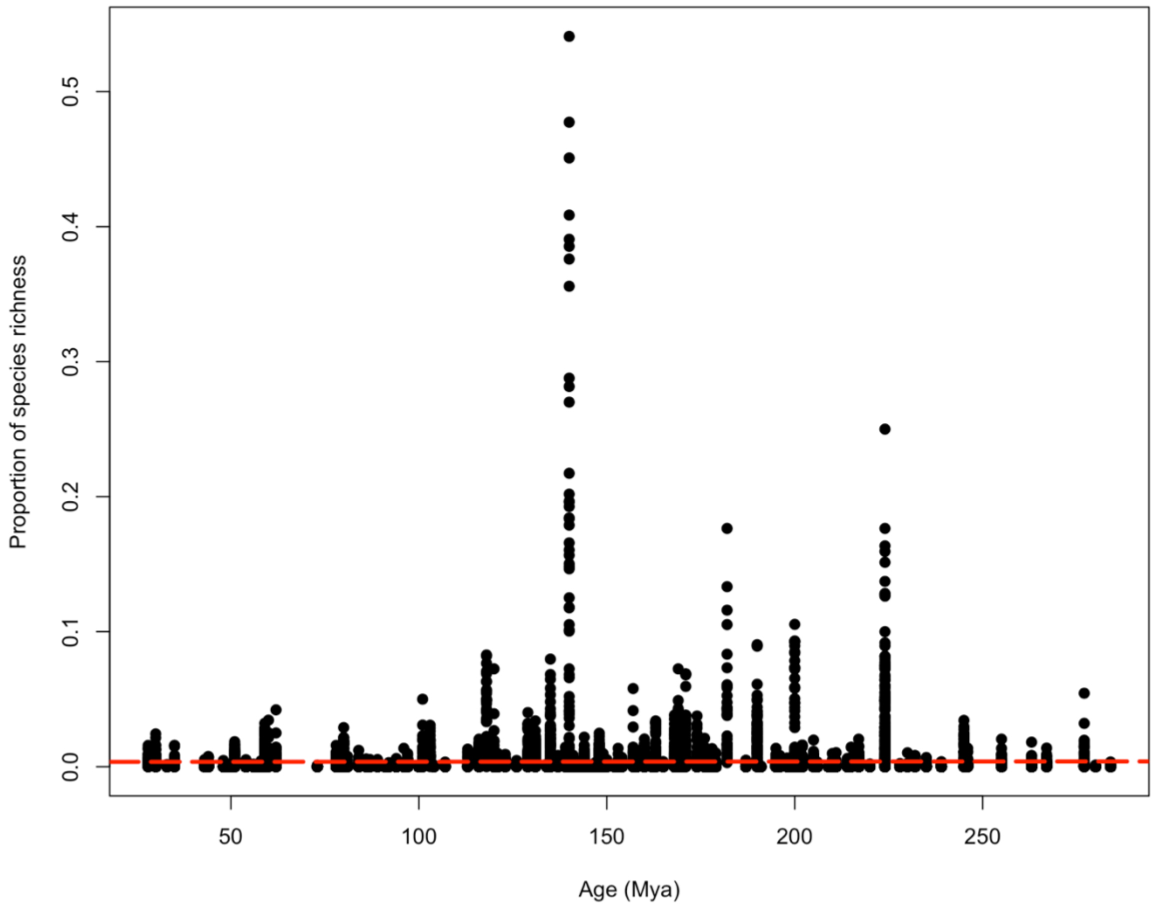

Supplementary Fig 5. Correlation between age of clade (Family) and proportion of species richness in the trap

Table 1: Number of mOTUs based on different species delimitation methods and distance thresholds. Column 1 specifies the trap ID, column 2 provides the number of specimens with barcodes, and 3 provides the number of specimens used in the analysis after non-flying insects and specimens unidentified to family. Columns 4 onwards provides number of mOTUs obtained with different species delimitation algorithms. OC1/2/3% show results of objective clustering at 1, 2, and 3%. ASAP1,2,3 show results of ASAP partition with best, 2nd best and 3rd best partitioning schemes. Corresponding values after removing non-flying insects and species unidentified to families are provided in Supplementary Tables 6 (OC3% main dataset), 9 (OC3%, expanded dataset), and 12 (ASAP1, main dataset). PTP column represents species delimitations by PTP. Final column presents the number of OC3% species unidentified to family.

**Trap**	**#specimens**	**#specimens after removing non flying insects**	**OC2%**	**OC3%**	**OC4%**	**ASAP1**	**ASAP2**	**ASAP3**	**PTP**	**%OC3% species unidentified to family**
CON03	147	133	79	79	78	78	78	83	80	9
CON05	353	339	116	114	112	112	99	114	119	10
KM03	1544	1432	432	425	420	411	417	409	431	29
KM04	8533	8194	1282	1247	1227	1180	1112	1211	1271	78
KM05	1623	1455	419	409	404	411	412	407	413	20
MISL02	1150	1061	636	628	622	620	623	615	631	36
MISL03	3156	2705	706	700	695	680	687	668	706	47
MISL06	988	816	419	408	403	403	396	402	407	21
MISL07	2386	750	396	390	386	376	2	410	402	33
MISL10	2075	1879	574	560	550	551	535	557	566	21
PU01	1418	1317	528	518	516	509	508	502	473	46
PU22	383	358	205	201	198	197	182	200	225	13
PU23	288	273	133	132	130	130	132	129	133	11
PU24	545	493	222	218	215	214	213	209	221	22
PU25	1193	1127	360	359	355	350	360	354	360	31
PU27	554	493	250	247	245	245	247	243	249	22
PU29	369	268	171	170	169	173	169	172	173	19
PU26	4271	3193	984	971	959	903	903	925	983	75
12-Sep-12	1572	1515	483	469	459	441	446	445	503	
15-Aug-12	1131	1022	374	368	355	329	338	339	375	
18-Jul-12	1273	1195	380	372	363	359	360	380	390	
1-Aug-12	814	774	251	246	240	239	223	241	249	
20-Jun-12	2440	2345	635	619	606	587	593	584	648	
23-May-12	2275	2216	451	442	431	434	430	423	469	
29-Aug-12	1685	1585	419	410	397	380	378	417	427	
4-Jul-12	2111	2011	611	589	569	542	542	606	611	
6-Jun-12	1931	1862	496	485	472	477	461	478	505	
9-May-12	3795	3710	453	438	418	393	388	376	457	

DeWaard et al (all)	27935*	27143*	2211	2121	2012	1888	1894	1894	NA	4
Canada Site3 (Telfer et al.)	2673	2550	925	901	880	883	847	886	941	1
20-Jun-12	3194	3070	793	773	749	763	755	752	810	
22-Aug-12	4529	4446	815	786	760	777	763	764	825	
22-May-12	1691	1617	347	339	333	335	338	335	349	
22-Sep-12	720	704	253	249	244	246	243	242	252	
25-Jul-12	5094	5034	864	835	809	818	815	822	882	
3-Sep-12	2366	2329	452	442	429	439	423	459	462	
4-Jul-12	2234	2215	636	618	607	633	626	620	646	
8-Jun-12	2531	2460	549	533	523	526	522	528	555	
8-May-12	1646	1624	242	239	233	238	235	232	242	
Geiger et al. (All GMTPE)	24005	23499	2502	2397	2284	2316	2337	2336	NA	25
TrapC	7437	6429	2048	1947	1862	1846	3025	1793	2034	197
TrapE	8546	7978	2262	2190	2125	2207	2103	2102	2279	188
TrapI	6662	6058	2148	2077	2016	2009	2009	2022	2180	168
TrapJ	11576	10466	2987	2885	2817	2815	2804	2822	3006	334
TrapO	6768	6045	1859	1791	1738	1588	2843	2452	1858	207
TrapP	4923	4291	1229	1197	1166	1136	1132	1126	1229	112
TrapU	20573	19107	4036	3888	3727	3883	3890	3957	4108	374
TrapW	21423	19766	3400	3271	3162	NA	NA	NA	3458	276
TrapX	10684	9343	2857	2750	2654	2553	2677	2531	2907	251
ALEXAN-1	5788	5780	568	561	554	562	564	565	579	1
ALEXAN-2	8646	8641	625	617	613	613	613	620	627	2
SaudiArabia Trap1	8691	8685	678	659	650	659	659	609	691	1
Pakistan Museum of Natural History	17901	17900	2199	2143	2088	2129	2018	2112	2262	1
Base Camp	10409	7279	1986	1935	1895	1879	1876	1866	1993	33
Cantiles	4308	3103	1094	1066	1044	1060	1039	1064	1104	24
Cortecito	1304	1169	691	682	674	676	676	671	689	8
Danto	818	602	346	341	339	335	346	359	346	7
GU	14460	12049	3509	3421	3358	3367	3375	3370	3540	48

*Includes counts of Chironomidae morphospecies that were not sequenced (Morphospecies 1: 8595 specimen, Morphospecies 2: 313)

Table 2: Number of species in the top 20 most common insect families that were found in a single vs at multiple sites.

**Family**	**# species in one site**	**# species in multiple sites**	**Total**
Diptera: Cecidomyiidae	4839	48	4887
Hymenoptera: Platygastridae	1466	44	1510

Diptera: Chironomidae	952	16	968
Diptera: Phoridae	907	37	944
Hymenoptera: Ichneumonidae	887	16	903
Hymenoptera: Braconidae	842	15	857
Diptera: Sciaridae	819	26	845
Diptera: Ceratopogonidae	737	16	753
Hemiptera: Cicadellidae	610	16	626
Hymenoptera: Formicidae	605	10	615
Hymenoptera: Eulophidae	564	9	573
Hymenoptera: Bethylidae	393	5	398
Lepidoptera: Erebidae	371	20	391
Diptera: Mycetophilidae	307	6	313
Hemiptera: Aleyrodidae	292	9	301
Diptera: Chloropidae	269	31	300
Diptera: Psychodidae	285	12	297
Diptera: Muscidae	262	26	288
Diptera: Dolichopodidae	240	7	247
Diptera: Sphaeroceridae	191	16	207
Lepidoptera: Crambidae	116	8	124

Table 3: Robustness of list of top 20 families after the merger of families in top 21–30 with sister clades. If sister clade was not in the dataset or the group is not monophyletic, taxon was merged with all families forming a monophyletic clade. Old rank is the rank of top 21–30 families (highlighted in bold) prior to merger with sister clades. References used are provided below the table.

New rank	Old rank	Taxon
1	1	Diptera: Cecidomyiidae
2	2	Diptera: Ceratopogonidae
3	3	Diptera: Chironomidae
4	4	Hymenoptera: Platygastridae
5	5	Diptera: Psychodidae
6	6	Diptera: Sciaridae
7	7	Diptera: Phoridae
8	8	Hymenoptera: Formicidae
9	9	Hemiptera: Cicadellidae
10	10	Hymenoptera: Ichneumonidae
11	11	Hymenoptera: Braconidae
12	12	Diptera: Dolichopodidae
13	13	Diptera: Muscidae
14	30	Lepidoptera: **Gelechiidae** + Cosmopterigidae[1,2]
15	14	Lepidoptera: Erebidae
16	15	Diptera: Sphaeroceridae

17	16	Hemiptera: Aleyrodidae
18	17	Hymenoptera: Eulophidae
19	18	Hymenoptera: Bethylidae
20	19	Diptera: Chloropidae
21	20	Lepidoptera: Crambidae
22	21	Coleoptera: **Staphylinidae** + Silphidae + Leiodidae + Agyrtidae[3]
23	23	Diptera: **Mycetophilidae** + Lygistorrhinidae + Keroplatidae + Bolitophilidae + Ditomyiidae + Diadocidiidae[4**]
24	22	Diptera: **Limoniidae** + Tipulidae + Cylindrotomidae[5]
25	27	Coleoptera: **Chrysomelidae** + Cerambycidae + Megalopodidae[3]
26	24	Coleoptera: **Curculionidae** + Brentidae[3]
27	25	Psocoptera: **Lepidopsocidae** + Trogiidae + Psoquillidae[6]
28	26	Diptera: **Drosophilidae** + Cryptochetidae + Braulidae[7]
29	28	Hymenoptera: **Mymaridae** (sister to rest of Chalcicoidea, including Eulophidae (Rank 17))[8,9]
30	29	Diptera: **Tachinidae** + Polleniidae[10]

****** Alternate hypotheses by Ševčík et al. (2016)[11] would group Mycetophilidae with a clade containing Sciaridae, which is in the top-10 families (Rank 6)

1. Sohn, J.-C. et al. Phylogeny and feeding trait evolution of the mega-diverse Gelechioidea (Lepidoptera: Obtectomera): new insight from 19 nuclear genes. *Systematic Entomology* **41**, 112–132 (2016).
2. Wang, Q. & Li, H. Phylogeny of the superfamily Gelechioidea (Lepidoptera: Obtectomera), with an exploratory application on geometric morphometrics. *Zoologica Scripta* **49**, 307–328 (2020).
3. Cai, C. et al. Integrated phylogenomics and fossil data illuminate the evolution of beetles. *Royal Society Open Science* **9**, doi:10.1098/rsos.211771 (2022).
4. de Souza Amorim, D. & Rindal, E. Phylogeny of the Mycetophiliformia, with proposal of the subfamilies Heterotrichinae, Ohakuneinae, and Chiletrichinae for the Rangomaramidae (Diptera, Bibionomorpha). *Zootaxa* **1535**, 1–92 (2007).
5. Peterson, M. J., Bertone, M. A., Wiegmann, B. M. & Courtney, G. W. Phylogenetic synthesis of morphological and molecular data reveals new insights into the higher-level classification of Tipuloidea (Diptera). *Systematic Entomology* **35**, 526–545 (2010).
6. Yoshizawa, K., Lienhard, C. & Johnson, K. P. Molecular systematics of the suborder Trogiomorpha (Insecta: Psocodea: 'Psocoptera'). *Zoological Journal of the Linnean Society* **146**, 287–299 (2006).
7. Bayless, K. M. et al. Beyond Drosophila: resolving the rapid radiation of schizophoran flies with phylotranscriptomics. *BMC Biology* **19**, 23 (2021).
8. Peters, R. S. et al. Transcriptome sequence-based phylogeny of chalcidoid wasps (Hymenoptera: Chalcidoidea) reveals a history of rapid radiations, convergence, and evolutionary success. *Molecular Phylogenetics and Evolution* **120**, 286–296 (2018).
9. Munro, J. B. et al. A Molecular phylogeny of the Chalcidoidea (Hymenoptera). *PLoS ONE* **6**, e27023 (2011).
10. Yan, L. et al. Monophyletic blowflies revealed by phylogenomics. *BMC Biology* **19**, 230 (2021).

11. Ševčík, J. et al. Molecular phylogeny of the megadiverse insect infraorder Bibionomorpha *sensu lato* (Diptera). *PeerJ* **4**, e2563 (2016).

Supplementary Material 1

Estimation of true global insect species diversity

Stork et al. (2015) use the Ratio between butterfly species diversity and insect diversity in UK based on Barnard (2011). This is extrapolated to 15000-20000 butterfly species present globally. Thus the estimate of 5.4-7.2 million species million is derived from $\frac{24043}{67} \times 15{,}000$ and $\frac{24043}{67} \times 20{,}000$.

Based on Barnard (2011), overall 652 Cecidomyiidae species are described in the UK, i.e.

$$\frac{652}{24043} = 0.027 \text{ or } 2.7\%$$

On the other hand, based on current study, we estimate Cecidomyiidae diversity to be 19.98%. Assuming x Cecidomyiidae species are unknown to us, the total number of Cecidomyiidae species in UK would be $x + 652$. Inclusion of x species of Cecidomyiidae will also increase the total insect diversity to $x + 24043$.

Therefore,

$$\frac{x + 652}{x + 24043} = 0.1998$$

Solving this gives $x = 5188.44$

This estimates that Britain has approximately 5188 unknown Cecidomyiidae species, taking overall diversity to $x + 24043 = 29231$ species.

Assuming Lepidoptera diversity remains the same:

Minimum species diversity: $\frac{29231}{67} \times 15{,}000$ = 6.5 million

Maximum species diversity: $\frac{29231}{67} \times 20{,}000$ = 8.7 million

This takes the estimated species diversity to 6.5-8.7 million species.
