# Supplementary material for: Convergence of dominance and neglect in flying insect diversity
Source: Nat Ecol Evol. 2023 May 18;7(7):1012–21. doi: 10.1038/s41559-023-02066-0 (PMC10333119; doi:10.1038/s41559-023-02066-0)
Supplement: Supplementary file 2 — Reporting Summary [file 41559_2023_2066_MOESM2_ESM.pdf]

nature portfolio

Corresponding author(s): Rudolf Meier

Last updated by author(s): Mar 29, 2023

# Reporting Summary

Nature Portfolio wishes to improve the reproducibility of the work that we publish. This form provides structure for consistency and transparency in reporting. For further information on Nature Portfolio policies, see our Editorial Policies and the Editorial Policy Checklist.

## Statistics

For all statistical analyses, confirm that the following items are present in the figure legend, table legend, main text, or Methods section.

| n/a | Confirmed | |
|---|---|---|
| ☐ | ☒ | The exact sample size (*n*) for each experimental group/condition, given as a discrete number and unit of measurement |
| ☐ | ☒ | A statement on whether measurements were taken from distinct samples or whether the same sample was measured repeatedly |
| ☐ | ☒ | The statistical test(s) used AND whether they are one- or two-sided<br>*Only common tests should be described solely by name; describe more complex techniques in the Methods section.* |
| ☐ | ☒ | A description of all covariates tested |
| ☐ | ☒ | A description of any assumptions or corrections, such as tests of normality and adjustment for multiple comparisons |
| ☐ | ☒ | A full description of the statistical parameters including central tendency (e.g. means) or other basic estimates (e.g. regression coefficient) AND variation (e.g. standard deviation) or associated estimates of uncertainty (e.g. confidence intervals) |
| ☐ | ☒ | For null hypothesis testing, the test statistic (e.g. *F*, *t*, *r*) with confidence intervals, effect sizes, degrees of freedom and *P* value noted<br>*Give P values as exact values whenever suitable.* |
| ☒ | ☐ | For Bayesian analysis, information on the choice of priors and Markov chain Monte Carlo settings |
| ☒ | ☐ | For hierarchical and complex designs, identification of the appropriate level for tests and full reporting of outcomes |
| ☒ | ☐ | Estimates of effect sizes (e.g. Cohen's *d*, Pearson's *r*), indicating how they were calculated |

*Our web collection on statistics for biologists contains articles on many of the points above.*

## Software and code

Policy information about availability of computer code

| | |
|---|---|
| Data collection | Newly generated data for obtained for specimens collected using Malaise traps in Singapore. Data from other regions was obtained from published studies cited in the manuscript: Geiger et al (2016): 10.3897/BDJ.4.e10671; Telfer et al. (2015): 10.3897/BDJ.3.e6313; D'Souza et al (2021): 10.1016/j.biocon.2021.109034; D'Souza et al. (2018): 10.1111/mec.14693; DeWaard et al. (2018): 10.1139/gen-2018-0093; Ashfaq et al. (2017): 10.1371/journal.pone.0174749. For body size information was obtained from Rainford et al. (2016): 10.1186/s12862-015-0570-3. Other sources of information include Zoological Record (Web of Science), Catalogue of Life (https://creativecommons.org/licenses/by/4.0/ ), TimeTree (http://www.timetree.org/) |
| Data analysis | Statistical analyses in R v4.1.2, Species delimitation using: obj_cluster: https://github.com/asrivathsan/obj_cluster commit ID: 7d4d797, USEARCH (v 11.0.667), ASAP (source code downloaded on 20 September 2021 from https://bioinfo.mnhn.fr/abi/public/asap/last.tgz , RaxML (v8.2.5), mPTP (v0.2.4), Custom scripts: https://github.com/asrivathsan/malaisetraps. Maps plots for supplementary figures were built using ggmap v3.0.0 (R package) using tiles from Stamen Design and data OpenStreetMap contributors. |

For manuscripts utilizing custom algorithms or software that are central to the research but not yet described in published literature, software must be made available to editors and reviewers. We strongly encourage code deposition in a community repository (e.g. GitHub). See the Nature Portfolio guidelines for submitting code & software for further information.

## Data

Policy information about availability of data

All manuscripts must include a data availability statement. This statement should provide the following information, where applicable:

- Accession codes, unique identifiers, or web links for publicly available datasets
- A description of any restrictions on data availability
- For clinical datasets or third party data, please ensure that the statement adheres to our policy

The data is deposited to DOI: 10.6084/m9.figshare.20449401

## Human research participants

Policy information about studies involving human research participants and Sex and Gender in Research.

| | |
|---|---|
| Reporting on sex and gender | *Use the terms sex (biological attribute) and gender (shaped by social and cultural circumstances) carefully in order to avoid confusing both terms. Indicate if findings apply to only one sex or gender; describe whether sex and gender were considered in study design whether sex and/or gender was determined based on self-reporting or assigned and methods used. Provide in the source data disaggregated sex and gender data where this information has been collected, and consent has been obtained for sharing of individual-level data; provide overall numbers in this Reporting Summary. Please state if this information has not been collected. Report sex- and gender-based analyses where performed, justify reasons for lack of sex- and gender-based analysis.* |
| Population characteristics | *Describe the covariate-relevant population characteristics of the human research participants (e.g. age, genotypic information, past and current diagnosis and treatment categories). If you filled out the behavioural & social sciences study design questions and have nothing to add here, write "See above."* |
| Recruitment | *Describe how participants were recruited. Outline any potential self-selection bias or other biases that may be present and how these are likely to impact results.* |
| Ethics oversight | *Identify the organization(s) that approved the study protocol.* |

Note that full information on the approval of the study protocol must also be provided in the manuscript.

# Field-specific reporting

Please select the one below that is the best fit for your research. If you are not sure, read the appropriate sections before making your selection.

☐ Life sciences ☐ Behavioural & social sciences ☒ Ecological, evolutionary & environmental sciences

For a reference copy of the document with all sections, see nature.com/documents/nr-reporting-summary-flat.pdf

# Ecological, evolutionary & environmental sciences study design

All studies must disclose on these points even when the disclosure is negative.

| | |
|---|---|
| Study description | This study characterizes compositions of insect communities in Malaise trap samples and finds the 20 families that dominate the samples. It furthermore characterizes the taxonomic neglect for these dominant taxa. |
| Research sample | Samples obtained using Malaise traps which is a commonly used standardized insect sampling technique. All insects collected from the trap were analysed irrespective of sex. Newly generated data as well as published data is used. Published dataset corresponds to studies by Geiger et al (2016): 10.3897/BDJ.4.e10671; Telfer et al. (2015): 10.3897/BDJ.3.e6313; D'Souza et al (2021): 10.1016/j.biocon.2021.109034; D'Souza et al. (2018): 10.1111/mec.14693; DeWaard et al. (2018): 10.1139/gen-2018-0093; Ashfaq et al. (2017): 10.1371/journal.pone.0174749. Newly generated data as well as data obtained from the studies has been shared in doi provided in data availability statement |
| Sampling strategy | Sampling was conducted using malaise traps from various habitats and covering different continents and biogeographic regions. All specimens in the trap were sampled |
| Data collection | Data generated in this study or obtained from published studies. |
| Timing and spatial scale | Data generated from the study were collected between 3 May 2019 to 9 May 2019 from various habitats in Singapore. |
| Data exclusions | We excluded barcode sequences that contained a stop codon when translated using the invertebrate mitochondrial genetic code, or which could not be identified to family. Analyses were limited to insects (i.e., spiders, Collembola etc were excluded: see list in Supplementary Table 14 and 15). |

| | |
|---|---|
| Reproducibility | Multiple species delimitation methods were tested. Effect of smaller number of traps in some regions was assessed by using expanded datasets which uses sample level resolution. |
| Randomization | Randomization is not relevant because we used existing data as well as newly generated data. |
| Blinding | Blinding is not relevant to a study involving species delimitation using DNA barcodes and community composition analysis. |

Did the study involve field work? ☒ Yes ☐ No

## Field work, collection and transport

| | |
|---|---|
| Field conditions | Malaise traps samples were collected between 3 May 2019 to 9 May 2019 (Mean temperature for May 2019: ~29 °C; Total rainfall for May 2019: ~70mm) |
| Location | Newly generated data collected from Singapore from following latitudes,longitudes CON03: (1.409583,103.923028); CON05 (1.409444, 103.923028); KM03: (1.42, 103.730833); KM04: (1.419722, 103.73175); KM05: (1.4195, 103.731528); MIS-L02: (1.410139, 103.784444); MIS-L03: (1.406722, 103.788222); MIS-L06: (1.405694, 103.785083); MIS-L07: (1.406111, 103.784639); MIS-L10: (1.405361, 103.782278); PU01: (1.419889, 103.935084); PU22: (1.418139, 103.935139); PU23: (1.426972, 103.935056); PU24: (1.426972, 103.9355); PU25: (1.418472, 103.941444); PU26: (1.426306, 103.936556); PU27: (1.418389, 103.941222); PU29: (1.41975, 103.935139). |
| Access & import/export | Sampling was conducted with permits and assistance from the National Biodiversity Centre of NParks and the Mandai Park Holding (Permits: NP/RP12-022-4, NP/RP12-022-5, NP/RP12-022-6). |
| Disturbance | Malaise traps are stationed passively on one location in the habitat, and the collection only requires weekly retrieval of bottle with ethanol and insects. |

# Reporting for specific materials, systems and methods

We require information from authors about some types of materials, experimental systems and methods used in many studies. Here, indicate whether each material, system or method listed is relevant to your study. If you are not sure if a list item applies to your research, read the appropriate section before selecting a response.

### Materials & experimental systems

| n/a | Involved in the study | |
|---|---|---|
| ☒ | ☐ | Antibodies |
| ☒ | ☐ | Eukaryotic cell lines |
| ☒ | ☐ | Palaeontology and archaeology |
| ☐ | ☒ | Animals and other organisms |
| ☒ | ☐ | Clinical data |
| ☒ | ☐ | Dual use research of concern |

### Methods

| n/a | Involved in the study | |
|---|---|---|
| ☒ | ☐ | ChIP-seq |
| ☒ | ☐ | Flow cytometry |
| ☒ | ☐ | MRI-based neuroimaging |

## Animals and other research organisms

Policy information about studies involving animals; ARRIVE guidelines recommended for reporting animal research, and Sex and Gender in Research

| | |
|---|---|
| Laboratory animals | Study did not involve laboratory animals |
| Wild animals | Insects were collected using Malaise traps in Singapore using vials of ethanol. The animals were killed when they entered ethanol, and this is a standard procedure for collection of insect samples. |
| Reporting on sex | Study does not involve characterization of sex. |
| Field-collected samples | Ethanol preserved insects were kept in room temperature prior to DNA extraction and PCR |
| Ethics oversight | No ethical approval is required as the sampling was conducted with permits from authorities using standardized insect sampling techniques that kills the insects. |

Note that full information on the approval of the study protocol must also be provided in the manuscript.
